# Operationalization of rapid molecular test coverage for tuberculosis in different international contexts: a scoping review

**DOI:** 10.1590/1980-220X-REEUSP-2026-0172en

**Published:** 2026-08-10

**Authors:** Diogo Henrique Mendes da Silva, Ana Beatriz Marques Valença, Melisane Regina Lima Ferreira, Mariana Gaspar Botelho Funari de Faria, Rubia Laine de Paula Andrade, Rebeca Sousa Braga, Aline Aparecida Monroe

**Affiliations:** 1 Universidade de São Paulo, Escola de Enfermagem de Ribeirão Preto, Programa de Pós-Graduação Enfermagem em Saúde Pública, Ribeirão Preto, SP, Brazil.; 2 Ministério da Saúde, Secretaria de Vigilância em Saúde e Ambiente, Coordenação-Geral de Vigilância da Tuberculose, Micoses Endêmicas e Micobactérias Não Tuberculosas, Brasília, DF, Brazil.; 3 Universidade de São Paulo, Escola de Enfermagem de Ribeirão Preto, Departamento de Enfermagem Materno-Infantil e Saúde Pública, Ribeirão Preto, SP, Brazil.

**Keywords:** Tuberculosis, Molecular Diagnostic Techniques, Rapid Diagnostic Tests.

## Abstract

**Objective:**

To map studies on the coverage of the rapid molecular test for tuberculosis in different international contexts.

**Method:**

A scoping review conducted according to the PRISMA-ScR recommendations, with searches in the MEDLINE, Scopus, Web of Science, Embase, LILACS, and BDTD databases, covering the period from 2011 to 2025.

**Results:**

The 20 included studies revealed conceptual heterogeneity in the operationalization of coverage, ranging from proportions of testing in specific population groups to indicators of equipment availability and geographic accessibility, with no standardization across countries and contexts. Values ranged from less than 1% to approximately 80%, with marked differences across countries, target populations, and analyzed periods. Socioeconomic, structural, financial, and facility-access challenges hinder the use of the test.

**Conclusion:**

Test coverage varies across countries and contexts, limited by structural and financial barriers and by the lack of conceptual and methodological standardization in its measurement. Expanding and monitoring coverage in a comparable way requires governmental commitment, decentralization, and the adoption of uniform criteria aligned with World Health Organization guidelines.

## INTRODUCTION

Although curable, tuberculosis (TB) still poses a challenge to health systems worldwide. The high number of people who fall ill in developing countries reinforces the need to sustain prevention and control strategies^(1)^. In 2025, it is estimated that more than 10 million people continue to fall ill with TB every year and that more than one million die from the disease^(2)^.

Sputum smear microscopy, although a simple and low-cost diagnostic technique, has limitations such as low sensitivity and specificity^(3,4)^. Initially, these limitations were overcome by performing microbiological cultures, considered the reference standard for the diagnosis of TB, drug-resistant TB (DR-TB), and nontuberculous mycobacteria^(3)^. However, the main disadvantage of culture is the prolonged time to obtain results, usually between four and eight weeks^(5)^.

Owing to these limitations, new diagnostic methods were developed to improve disease detection and to advance toward the Sustainable Development Goals (SDGs), adopted in September 2015, related to the social and health response to the fight against TB^(6)^.

In this regard, among the molecular tests developed, GeneXpert MTB/RIF technology, known in Brazil as the Rapid Molecular Test for Tuberculosis (RMT-TB), was recommended worldwide by the World Health Organization (WHO) starting in December 2010^(5)^, and was later improved and replaced by Xpert MTB/RIF Ultra in 2019, a version with greater sensitivity and specificity^(7)^. Both tests are capable of detecting the *Mycobacterium tuberculosis* complex and identifying rifampicin-resistant strains in biological samples in approximately two hours^(3,4,7)^.

After this recommendation, several countries began to adopt the technology to diagnose new cases of TB. Nevertheless, studies conducted under routine care conditions show that the RMT-TB increased case detection and improved access to diagnosis^(5,8)^.

Despite the recommendations for implementing the test in different contexts, gaps persist not only regarding its practical application in health systems but, above all, regarding how its coverage is defined and measured. The absence of standardized criteria to operationalize RMT-TB coverage hampers comparisons between countries and the assessment of progress toward the targets set by the WHO, constituting a scientific gap that this review proposes to map. To understand this gap, coverage, access, utilization, and availability are distinct concepts, although frequently used in an overlapping manner in the literature. Coverage, in a broad sense, expresses the degree of interaction between the service and the population it is intended to serve, from the allocation of resources to the attainment of the intended outcome^(9)^.

In view of this, the objective of this study is to map studies on RMT-TB coverage in different international contexts, identifying how this indicator has been operationalized and described in the literature, in order to inform management strategies and health policies aimed at TB control.

## METHOD

### Study Design

This is a scoping review carried out following the recommendations of the Preferred Reporting Items for Systematic reviews and Meta-Analyses extension for Scoping Reviews (PRISMA-ScR) checklist^(10)^ to guide the reporting of the results. It was conducted according to the JBI manual^(11)^, which establishes the methodological stages of a scoping review: 1) defining the research question; 2) identifying relevant studies; 3) screening and preliminary assessment of the studies; 4) analyzing the information from the studies; and 5) organizing, synthesizing, and presenting the results. PRISMA-ScR was used as a guide for transparency in reporting each of these stages. The protocol of this review was registered in the Open Science Framework (OSF) under the DOI: https://doi.org/10.17605/OSF.IO/6EUH7.

### Data Collection

The guiding question “How has the coverage of the RMT-TB for the diagnosis of people with TB or with signs and symptoms suggestive of TB been operationalized and described in different international contexts?” was developed based on the Population, Concept, and Context (PCC) mnemonic methodology. In this model, the Population (P) was represented by people with TB or with signs and symptoms suggestive of TB; the Concept (C) corresponded to RMT-TB coverage; and the Context (C) encompassed the different international contexts.

Based on this question, this study adopted the broad conception of health service coverage proposed by Tanahashi, understood as the degree of interaction between the service and the population it is intended to serve, encompassing everything from the availability and allocation of resources to the attainment of the intended outcome^(9)^. Considering the dimensions of availability, accessibility, acceptability, contact, and effectiveness, the indicators of coverage considered were the proportion of tests performed in population groups, the availability of equipment in health services, and the geographic accessibility to the diagnostic facilities, according to the operationalization presented in each study. Such dimensions, considered as indicators of coverage, were identified through the reading of the included studies.

To identify the relevant studies, an electronic search was performed in the following databases: Scopus, Web of Science, Medical Literature Analysis and Retrieval System Online (MEDLINE), Latin American and Caribbean Health Sciences Literature (LILACS), and Embase. Searches were also performed to identify gray literature in the Brazilian Digital Library of Theses and Dissertations (BDTD).

The electronic search was conducted in December 2025 using keywords previously identified in the literature and descriptors indexed in the Health Sciences Descriptors (DeCS) platform, as well as their synonyms in Portuguese and Spanish. Descriptors indexed in the Medical Subject Headings (MeSH) platform were also used to develop the search strategies in English. To combine the descriptors and keywords with one another, the Boolean operators AND and OR were applied. The descriptors, keywords, period, and search strategies performed for each database are presented in Chart 1. The search strategy was validated by the coauthors, members of the same research group. The language filters (English, Portuguese, and Spanish) were applied at the study screening stage.

**Chart 1:** Strategies used to identify studies, applied to each database – Ribeirão Preto, SP, Brazil, 2025.

Databases	Search strategies
MEDLINE	*((“tuberculosis” [MeSH Terms] OR “tuberculosis” [All Fields] OR “tuberculoses” [All Fields] OR “tb” [All Fields]) AND (“xpert” [All Fields] OR “genexpert” [All Fields] OR “gene xpert” [All Fields] OR “cepheid” [All Fields] OR “cepheids” [All Fields] OR “rapid molecular test” [All Fields]) AND (“coverage” [All Fields] OR “coverages” [All Fields] OR “diagnostic coverage” [All Fields])) AND (medline[Filter]) AND (2011:2025[pdat])*
Scopus	*(TITLE-ABS-KEY (tuberculosis OR tb) AND TITLE-ABS-KEY (xpert OR genexpert OR “gene xpert” OR cepheid OR “rapid molecular test”) AND TITLE-ABS-KEY (coverage OR “diagnostic coverage” ) ) AND PUBYEAR > 2010 AND PUBYEAR < 2026*
Web of Science	*tuberculosis OR tb (Topic) and xpert OR genexpert OR “gene xpert” OR cepheid OR “rapid molecular test” (Topic) and coverage OR “diagnostic coverage” (Topic) and 2025 or 2024 or 2023 or 2022 or 2021 or 2020 or 2019 or 2018 or 2017 or 2016 or 2015 or 2014 or 2013 or 2012 or 2011 (Publication Years)*
Embase	*#1 (‘tuberculosis’/exp OR tuberculosis OR ‘tb’/exp OR tb) AND [2011–2025]/py #2 xpert OR genexpert OR ‘gene xpert’ OR cepheid OR ‘rapid molecular test’ #3 coverage OR ‘diagnostic coverage’ #4 #1 AND #2 AND #3*
LILACS	*(tuberculosis OR tuberculose OR tb) AND (xpert OR genexpert OR “gene xpert” OR cepheid OR “rapid molecular test” OR “teste rápido molecular” OR trm-tb OR “teste molecular rápido” OR “prueba rápida molecular” OR prm OR “prueba molecular rápida”) AND (coverage OR “diagnostic coverage” OR cobertura OR “cobertura diagnóstica” OR “cobertura de diagnóstico”) AND (year_cluster:[2011 TO 2025])*
Biblioteca Digital Brasileira de Teses e Dissertações	*“(Todos os campos:tuberculosis OR tuberculose OR tb E Todos os campos:xpert OR genexpert OR “gene xpert” OR cepheid OR “rapid molecular test” OR “teste rápido molecular” OR trm-tb OR “teste molecular rápido” OR “prueba rápida molecular” OR prm OR “prueba molecular rápida” E Todos os campos:coverage OR “diagnostic coverage” OR cobertura OR “cobertura diagnóstica” OR “cobertura de diagnóstico”)”*

LILACS: Latin American and Caribbean Health Sciences Literature.

### Selection Criteria

The eligibility criteria for this scoping review comprised studies that addressed the coverage or percentage of use of the RMT-TB (GeneXpert MTB/RIF and/or Xpert MTB/RIF Ultra), understood as quantitative indicators of use (coverage based on the use of the RMT-TB among people with signs and symptoms suggestive of TB; coverage based on the use of the RMT-TB among people diagnosed with TB; coverage based on the use of the RMT-TB in people with DR-TB; coverage based on the use of the RMT-TB in people living with HIV; and coverage based on the use of the RMT-TB among people who tested negative on sputum smear microscopy) and/or availability of or access to the test (coverage based on the number of RMT-TB machines relative to the number of health facilities; and RMT-TB coverage based on the population’s distance to diagnostic facilities). Thus, studies that presented these indicators as their main objective or as a secondary finding were included, provided that the quantitative data were clearly described; that had in the analyzed population people with TB or with signs and symptoms suggestive of TB, regardless of clinical form or vulnerability conditions; and that were published between 2011 and 2025.

The decision to consider these studies stems from their wide dissemination in national and international TB control programs, their recognition by the WHO as a reference for rapid molecular diagnosis, and the greater availability of studies adopting comparable metrics. This delimitation sought to reduce heterogeneity among distinct technologies and to allow greater consistency in the analysis of coverage. The choice of period is based on the recommendation adopted by the WHO for the use of the RMT-TB in TB and DR-TB testing, starting in December 2010^(2)^.

Studies published in English, Portuguese, and Spanish were eligible, whether primary or secondary, peer-reviewed or not, provided that their scope contemplated some indicator of RMT-TB coverage in any geographic area of the world, using any approach and methodological design. Theses, dissertations, undergraduate final papers, and conference abstracts were considered.

Protocols, manuals, guidelines, technical notes, letters, editorials, and commentaries were excluded, as they do not present empirical quantitative data on the use or coverage of the test that is the focus of this review. In addition, studies that did not present clear information related to coverage or to the RMT-TB in their title and abstract were discarded at the title and abstract screening stage. Studies that aggregate other types of molecular diagnostic tests distinct from GeneXpert MTB/RIF or Xpert MTB/RIF Ultra were also excluded, as they involve sample types and laboratory procedures not comparable to the RMT-TB defined for this review.

### Data Extraction and Analysis

After the database search process, the publications were exported to the Rayyan AI platform, a tool that assists review research in identifying and eliminating duplicates and selecting eligible studies^(12)^.

Extraction was carried out in two parts: the first was conducted by two reviewers independently, who assessed the titles and abstracts of the identified references, selecting potentially eligible studies based on the guiding question and the eligibility criteria. Disagreements were resolved by a qualified third author. The second part consisted of the full reading and assessment of the preselected studies, documenting the reasons for exclusion in a flowchart based on the PRISMA-ScR^(10)^.

The information from the included studies was extracted using an instrument adapted for use in an Excel spreadsheet containing the following variables: author(s); title; journal; type of publication; study language; country where the study was conducted; year of publication; objective; study design; study period; study population; sample size; operational definition of RMT-TB coverage; main results related to the coverage and/or percentage of use of the test; population for which the test is intended; and the authors’ main discussions and conclusions.

Of these variables, those directly related to the research question were presented: operational definition of coverage, measured coverage values, study design, and country where the study was conducted. The remaining variables were used to guide the narrative synthesis and the construction of the analytical categories.

All results were presented in the form of a descriptive chart containing the operational definition of coverage used and the coverage values measured in the studies. In addition, two categories of analysis were established to compose the discussion of the results: factors associated with low coverage and access to diagnosis through the RMT-TB. These categories emerged during the reading and analysis of the included studies, from the recurring themes identified in their discussions and conclusions.

### Ethical Aspects

This scoping review used exclusively secondary literature sources that are publicly available, without the collection of primary data or identifiable personal information. For this reason, and in accordance with the applicable ethical standards, approval by a Research Ethics Committee or the obtaining of an Informed Consent Form was not required.

## RESULTS

**Figure 1:**
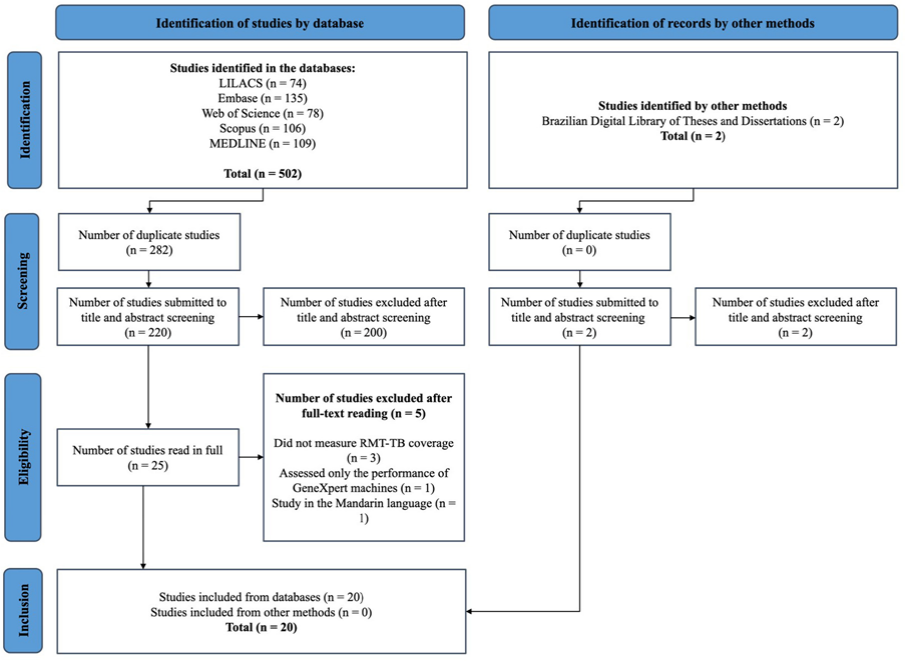
Flowchart of inclusion and exclusion of publications according to the PRISMA-ScR recommendations – Ribeirão Preto, Brazil, 2025. LILACS: Latin American and Caribbean Health Sciences Literature; RMT-TB: Rapid Molecular Test for Tuberculosis.

The included studies were analyzed with a focus on how RMT-TB coverage was operationalized and described in each context. A total of 502 records were identified in the databases. After the removal of duplicate studies, 220 were submitted to title and abstract reading. After this reading, the present review included 20 studies that met the inclusion criteria and answered the research question. The reasons for excluding articles read in full were documented as shown in the flowchart in Figure 1.

The publication period of the included studies ranged from 2017 to 2025^(13,14,15,16,17,18,19,20,21,22,23,24,25,26,27,28,29,30,31,32)^, with the highest concentration in 2020^(19,20,21)^, 2021^(22,23,24)^, and 2024^(28,29,30)^, years in which three publications each were identified. Regarding the language of publication, 19 studies were available in English^(13,14,15,16,17,18,19,21,22,23,24,25,26,27,28,29,30,31,32)^ and one in Spanish^(20)^. As for the study design, a predominance of quantitative approaches was observed, with only one study involving mixed methods^(22)^. Most were cohort studies^(16,17,19,24)^ (Chart 2). Among the included studies, seven distinct ways of operationalizing RMT-TB coverage were identified, categorized from the available information, namely: 1) coverage based on the use of the RMT-TB among people with signs and symptoms suggestive of TB (n = 8)^(15,17,18,24,26,27,29,30)^; 2) coverage based on the use of the RMT-TB among people diagnosed with TB (n = 4)^(13,14,19,20)^; 3) coverage based on the use of the RMT-TB in people with DR-TB (n = 2)^(16,31)^; 4) coverage based on the use of the RMT-TB in people living with HIV (n = 2)^(28,32)^; 5) coverage based on the use of the RMT-TB among people who tested negative on sputum smear microscopy (n = 2)^(21,23)^; 6) coverage based on the number of RMT-TB machines relative to the number of health facilities (n = 1)^(22)^; and 7) RMT-TB coverage based on the population’s distance to diagnostic facilities (n = 1)^(25)^ (Chart 2).

**Chart 2:** Presentation of the studies included in the review – Ribeirão Preto, SP, Brazil, 2025.

Author, year, and country	Publication type	Study design	Study objectives	Operational definition of coverage	Rapid molecular test coverage values
Rees et al., 2017^(13)^; South Africa	Article	Retrospective study	To assess diagnostic trends in patients treated for TB in a rural subdistrict of South Africa, in relation to the implementation of the RMT-TB and its accompanying algorithm, using routine data.	Coverage based on the use of the RMT-TB among people diagnosed with TB.	From 0% in 2010 to 69.9% in 2015 in the Greater Tzaneen subdistrict, South Africa.
Zemlyansky et al., 2017^(14)^; Russia	Article	Cross-sectional study	To assess effectiveness and develop an optimal model for the laboratory diagnosis of tuberculosis using the RMT-TB.	Coverage based on the use of the RMT-TB among people diagnosed with TB.	75.3% among new cases and 80.8% in relapses in the Belgorod region, Russia, in 2015.
Awan et al., 2018^(15)^; Pakistan	Article	Cross-sectional study	To fill the gaps in the literature on the potential constraints in the implementation of the RMT-TB in programmatic settings with a high burden of DR-TB.	Coverage based on the use of the RMT-TB among people with signs and symptoms suggestive of TB.	44.3% between July 2013 and June 2015 in Karachi, Pakistan.
Evans et al., 2018^(16)^; South Africa	Article	Retrospective cohort study	To analyze the impact of the introduction of the RMT-TB and of treatment decentralization on linkage to care and on DR-TB treatment outcomes in Johannesburg, South Africa.	Coverage based on the use of the RMT-TB in people with DR-TB.	43.4% between July 2011 and June 2012 and 60.5% between July 2013 and June 2014, in Johannesburg, South Africa.
Farr et al., 2019^(17)^; Uganda	Article	Prospective cohort study	To characterize the extent to which TB patients in Uganda were receiving care in accordance with national and international guidelines.	Coverage based on the use of the RMT-TB among people with signs and symptoms suggestive of TB.	19.5% in 2017 across 24 health centers in Uganda.
Kabasakalyan et al., 2019^(18)^; Armenia	Article	Retrospective observational study	To assess the change in the laboratory diagnostic profile of *Mycobacterium tuberculosis* after the introduction of the RMT-TB.	Coverage based on the use of the RMT-TB among people with signs and symptoms suggestive of TB.	58% in the 2013–2017 period in Armenia.
Wu et al., 2020^(19)^; China	Article	Retrospective cohort study	To assess the effect of implementing the RMT-TB on the detection of pulmonary TB cases.	Coverage based on the use of the RMT-TB among people diagnosed with TB.	54% in the first half of 2018 in Shanghai, China.
Agredo and Osorio, 2020^(20)^; Colombia	Article	Descriptive study	To describe the coverage and fidelity of RMT-TB use in patients with pulmonary tuberculosis in a high-disease-burden city in Colombia.	Coverage based on the use of the RMT-TB among people diagnosed with TB.	10.35% between 2013 and 2019 in the municipality of Cali, Colombia.
Creswell et al., 2020^(21)^; Guatemala	Article	Programmatic evaluation study	To assess the impact of introducing the RMT-TB as a follow-up test after smear microscopy on the total number of pulmonary TB notifications.	Coverage based on the use of the RMT-TB among people who tested negative on sputum smear microscopy.	39.6% between April 2015 and March 2016 in Guatemala.
Ntinginya et al., 2021^(22)^; Uganda, Kenya, and Tanzania	Article	Quantitative and qualitative	To investigate the barriers and opportunities for maximizing the uptake and use of molecular diagnostics in routine health care settings.	Coverage based on the number of RMT-TB machines relative to the number of health facilities.	From September 2016 to December 2017, 74% in Uganda, 39% in Tanzania, and 42% in Kenya.
Li et al., 2021^(23)^; China	Article	Before-and-after cross-sectional evaluation study	To assess the impact of the China-Gates Tuberculosis Control Project Phase III on access to quality diagnosis and treatment for TB.	Coverage based on the use of the RMT-TB among people who tested negative on sputum smear microscopy.	0% in 2015, 16% in 2018 using the RMT-TB alone, and 34.4% in 2018 using the RMT-TB + culture, in 12 localities of three provinces of China: Zhejiang, Jilin, and Ningxia.
Turaev et al., 2021^(24)^; Uzbekistan	Article	Prospective cohort study	To assess RMT-TB coverage among people with presumptive TB, the factors associated with it, and the delays involved.	Coverage based on the use of the RMT-TB among people with signs and symptoms suggestive of TB.	24% in 2018 and 46% in 2019 in Uzbekistan.
Milaham et al., 2022^(25)^; Nigeria	Article	Retrospective ecological study	To investigate the spatial distribution of TB case notification rates, diagnoses, and coverage across the Local Government Areas.	Rapid molecular test coverage based on the population’s distance to diagnostic facilities.	49% of the population lived more than 20 km from a RMT-TB facility between 2017 and 2019 in Katsina State, Nigeria.
Ishengoma et al., 2023^(26)^; Tanzania	Published abstract	Descriptive study in conference proceedings	To assess the effectiveness of mobile clinics in Tanzania as a strategy to detect TB cases not diagnosed in hard-to-reach areas.	Coverage based on the use of the RMT-TB among people with signs and symptoms suggestive of TB	34.5% across 8 regions of Tanzania using mobile clinics.
Getahun et al., 2023^(27)^; Ethiopia	Article	Retrospective study	To review the available records at selected health facilities in Addis Ababa, Ethiopia, and to assess the impacts of the RMT-TB on the number of TB cases detected and on treatment outcome successes.	Coverage based on the use of the RMT-TB among people with signs and symptoms suggestive of TB.	71.1% between 2015 and 2018 in Addis Ababa, Ethiopia.
Faria et al., 2024^(28)^; Brazil	Article	Ecological study	To analyze the diagnosis of unconfirmed pulmonary tuberculosis before and after the implementation of the RMT-TB for people living with HIV in municipalities of the state of São Paulo, Brazil.	Coverage based on the use of the RMT-TB in people living with HIV	49.4% between 2010 and 2020 in the state of São Paulo, Brazil.
Pank et al., 2024^(29)^; Papua New Guinea	Article	Prospective implementation study	To assess the initial implementation of the Systematic Island-Wide Engagement & Elimination Project for TB in Daru using a Continuous Quality Improvement initiative.	Coverage based on the use of the RMT-TB among people with signs and symptoms suggestive of TB.	77.9% between May and December 2023 in Daru, Papua New Guinea.
Dakulala et al., 2024^(30)^; Papua New Guinea	Article	Retrospective study	To screen the population of Daru island using a mobile van equipped with RMT-TB machines and digital X-ray with computer-aided detection software for TB.	Coverage based on the use of the RMT-TB among people with signs and symptoms suggestive of TB.	79.7% between February and October 2018 and February and March 2019, in Daru, Papua New Guinea, using a mobile van.
Mok et al., 2025^(31)^; South Korea	Article	Retrospective cohort study	To assess the coverage of molecular drug susceptibility testing among patients with pulmonary DR-TB in South Korea and to identify factors influencing its lack of implementation.	Coverage based on the use of the RMT-TB in people with DR-TB.	22.1% of all patients, with 23.5% among new cases and 19.9% among previously treated patients in South Korea.
Motlhaoleng et al., 2025^(32)^; South Africa	Article	Descriptive study	To assess the Targeted Universal Testing for TB care cascade to describe its implementation within the continuum of care for people living with HIV in high-volume (≥6500 patients), medium-volume (3500 to <6500 patients), and low-volume (<3500 patients) health facilities	Coverage based on the use of the RMT-TB in people living with HIV	In the rural district of Zululand, high-volume facilities presented 44.4% in 2022 and 43.3% in 2023; medium-volume facilities, 43.1% and 32.0%; and low-volume facilities, 55.0% and 63.9%, respectively. In the Municipality of eThekwini (urban district), high-volume facilities recorded 22.3% in 2022 and 28.3% in 2023; medium-volume facilities, 20.5% and 17.1%; and low-volume facilities, 2.2% and 5.5%.

TB: Tuberculosis; RMT-TB: Rapid Molecular Test for Tuberculosis; DR-TB: Drug-Resistant Tuberculosis; HIV: Human Immunodeficiency Virus.

In terms of operationalization, the categories differ regarding the numerator, denominator, and target population considered. In the categories based on the use of the test, the numerator corresponded to the number of people tested with the RMT-TB, and the denominator varied according to the population group: people with suggestive signs and symptoms, people diagnosed with TB, people with DR-TB, people living with HIV, and people with a negative smear result. In the category based on equipment availability, the numerator corresponded to the number of machines installed and the denominator to the number of health facilities. In the category based on geographic accessibility, the numerator corresponded to the population residing beyond a given distance from a diagnostic facility and the denominator to the total population of the studied area.

Regarding the number of studies by country where they were conducted: South Africa (n = 3)^(13,16,32)^, China (n = 2)^(19,23)^, Papua New Guinea (n = 2)^(29,30)^; South Korea (n = 1)^(31)^, Armenia (n = 1)^(18)^, Uzbekistan (n = 1)^(24)^, Russia (n = 1)^(14)^, Pakistan (n = 1)^(15)^, Ethiopia (n = 1)^(27)^, Uganda (n = 1)^(17)^, Tanzania (n = 1)^(26)^, Nigeria (n = 1)^(25)^, Brazil (n = 1)^(28)^, Colombia (n = 1)^(20)^, and Guatemala (n = 1)^(21)^. There was also one study involving three African countries: Uganda, Kenya, and Tanzania (n = 1)^(22)^.

From the reading of the included studies, two analytical categories emerged to organize the main findings: factors associated with low coverage and access to diagnosis through the RMT-TB.

### Factors Associated with Low Coverage

In the studies that reported low RMT-TB coverage, barriers to the use of the test were identified, including socioeconomic and cultural factors and structural limitations of the health systems. Among these, the following stood out: low active case finding, insufficient sample quality, use of equipment restricted to a single work shift, errors in completing requests, underfunding of health care, management difficulties between managers and professionals, shortage of qualified human resources, and the absence of basic infrastructure such as water supply and electricity^(22,24,28,29,32)^.

Some studies reported the use of RMT-TB machines with only four modules, associated with a smaller number of people tested and lower detection, especially of DR-TB; in these contexts, experiences with 16-module equipment to expand testing capacity were described^(25,26)^.

It was also reported, in certain countries, that there was dependence on health budgets funded by external donors, with reports that economic fluctuations in these countries affected the continuity of the supply of diagnosis and treatment^(22)^. In addition, one study mentioned difficulties faced by TB programs in planning interventions and investments based on point estimates of the TB burden from the WHO, which are subject to annual variations and wide confidence intervals^(21)^.

### Access to Diagnosis Through the RMT-TB

Some studies described limitations in the population’s access to the units that offer diagnosis through the RMT-TB. In Katsina State, Nigeria, the distribution of facilities was considered uneven, with 100% of the population of two Local Government Areas (LGAs) and 75% of eight LGAs located more than 20 km from any diagnostic point, indicating a greater distance to be traveled by the people residing in these areas^(25)^. In addition, the difficulty of infrastructure and medical care in rural areas compared with urban areas was emphasized^(32)^.

In Uganda, Tanzania, and Kenya, the RMT-TB was not used universally for all people with signs and symptoms suggestive of TB; its indication was prioritized for people living with HIV or with prior contact with DR-TB. As of 2019, the recommendation in these countries came to encompass all people with signs and symptoms suggestive of TB^(22)^.

In the cohort conducted in Tashkent and Bukhara, Uzbekistan, regional differences in access to the test were observed. The proportion of people who did not undergo the RMT-TB was higher in Bukhara (73%; 95%CI: 69–76) than in Tashkent (28%; 95% CI: 25–30) (p < 0.001). In Bukhara, 51% of people with TB sought units without a diagnostic service, compared with 9% in Tashkent; moreover, 80% of Bukhara residents accessed peripheral rural units, whereas 88% of Tashkent residents were linked to units located in central areas^(24)^.

The study conducted in South Africa points to weaknesses in the use of the RMT-TB among people living with HIV in the districts of Zululand (rural) and eThekwini (urban), when considering high-volume (≥6500 patients), medium-volume (3500 to <6500 patients), and low-volume (<3500 patients) facilities. The most critical situation occurs in the urban context, especially in the medium- and low-volume facilities^(32)^.

In twelve localities of three provinces of China, between 2015 and 2018, an increase in access to quality diagnosis for TB was observed, related to the increased use of the RMT-TB and to the expansion of diagnostic services for people with a negative smear result^(23)^.

## DISCUSSION

From the survey of the publications, a larger number of studies conducted on the African and Asian continents was found, which may be related to the high number and incidence of TB in these locations. Geographically, most people who developed TB in recent years were in the regions of South-East Asia (45%), Africa (24%), and the Western Pacific (17%), with smaller proportions in the Eastern Mediterranean (8.6%), the Americas (3.2%), and Europe (2.1%)^(2)^.

A central finding of this review was the conceptual and operational heterogeneity of the term “coverage” associated with the RMT-TB. Although the WHO uses as its baseline criterion the proportion of people with TB initially tested with a RMT-TB^(2)^, only four of the included studies explicitly adopted this definition^(13,14,19,20)^. It was observed, however, that other studies assessed aspects related to test coverage, such as equipment availability, the proportion of testing in population subgroups, or access to diagnosis, without naming them as coverage measures.

The diversity observed in the studies reflects the breadth of the very concept of health service coverage which, in a broad sense, ranges from the availability of resources to the attainment of the intended outcome^(9)^, approaching, in some studies, concepts such as access and utilization.

This conceptual imprecision, although it does not undermine legitimate operationalizations, has direct implications for the comparability of the data: when some studies measure coverage as the proportion of people tested among respiratory symptomatics, others as equipment availability, and others as geographic accessibility, the results become incomparable with one another. The different results reflect distinct dimensions of the same phenomenon, and equating them hinders both the diagnosis of the situation and the assessment of the impact of interventions. The absence of a common conceptual framework to operationalize RMT-TB coverage therefore represents a structural limitation of the field that this review highlights.

This overlap is recognized in the health systems literature. Penchansky and Thomas define access as a multidimensional concept that encompasses availability, accessibility, affordability, accommodation, and acceptability^(33)^, dimensions that appear in a fragmented manner in the different operationalizations of coverage identified in this review, reinforcing the need for greater conceptual precision in the field.

Since December 2010, the WHO has recommended the use of the RMT-TB as the primary diagnostic method for TB^(2,31)^; however, it was observed that, in two studies, the use of the test was tied to a negative smear result. This may have affected the coverage result in those locations, since in Guatemala, where this criterion was used, the RMT-TB alone resulted in a 12.3% increase in pulmonary TB notifications^(21)^. Despite its wide adoption for TB diagnosis, smear microscopy has a relatively low sensitivity compared with the RMT-TB, which is able to provide results much more quickly and, consequently, allows the early institution of treatment^(3,4,14,32)^.

A study conducted in the state of São Paulo, Brazil, also indicated that the coverage of smear microscopy exceeded that of the RMT-TB among people living with HIV, reaching a coverage of 70%^(28)^; that is, even with the high coverage of the RMT-TB, smear microscopy has been used in a complementary manner to the test, which increases the number of underreported cases, especially among paucibacillary individuals such as people living with HIV^(5)^, and may also result in treatments being conducted without bacteriological confirmation.

It is known that the risk of falling ill with TB is 23 times higher in people living with HIV^(34)^; thus, the need to continue using the RMT-TB, aligned with robust strategies that strengthen active case finding and the follow-up of these people, is reinforced.

Based on these findings, the aforementioned challenges in access to diagnosis through the RMT-TB reflect structural and operational barriers in care services, especially for users coinfected with TB and HIV, which reinforces the need for context-specific strategies and quality-improvement interventions to optimize the implementation of the RMT-TB in this specific population.

The RMT-TB is also capable of detecting dead and/or inactive bacilli, and is therefore contraindicated for people undergoing TB retreatment^(2)^; however, in the study conducted in Russia, in Belgorod, in addition to diagnosing new cases, the RMT-TB was also used to detect disease relapses^(14)^, signaling a possible inappropriate use of resources, with potential repercussions for the management of services, programs, and public policies. Similar situations were observed in the study conducted in South Korea, where the use of the test in previously treated patients was reported^(31)^.

Regardless of the criterion adopted, RMT-TB coverage varied across the different study sites, with some of them showing positive changes over the years. The results corroborate what has been reported by the WHO, which in 2024 found substantial variation in rapid test coverage across regions and countries. Among regions, the best coverage levels were achieved in the European Region (77%) and the Western Pacific Region (70%), while the lowest coverage was identified in the South-East Asia Region (41%). With respect to countries, 69 achieved coverage levels of at least 80% in 2024, but in 21 countries coverage was below 20%^(2)^. It is worth noting that low coverage may be an indicator of underdiagnosed TB, especially DR-TB^(16,22)^.

The WHO End TB Strategy sets the goal of eliminating tuberculosis as a public health problem by 2035, with a 95% reduction in deaths and a 90% reduction in incidence compared with 2015^(35)^. The data show that achieving these goals depends not only on making the RMT-TB available but also on overcoming the structural, organizational, and conceptual barriers identified in this review.

Low RMT-TB coverage was associated mainly with socioeconomic and structural difficulties of the different health systems; in addition, a lack of national or regional guidelines for the implementation of molecular tests in TB programs around the world is observed^(20)^. In 2015, a study that monitored the implementation of the RMT-TB in 22 countries indicated that, at that time, only Brazil and Russia funded all tests with domestic resources, while most other countries depended to some degree on some of the 16 international donor groups identified^(36)^.

Although financial and material-resource support from third-party countries to enhance TB diagnosis should be encouraged, it is worth noting that maximizing the effects and long-term sustainability of the RMT-TB also depends on local government health leadership, strategic planning, coordination of technical partners and donors, and continuous monitoring and assessment. Large-scale implementation requires the adoption of a revision of national algorithms, policies, registries, request forms, and monitoring and assessment methods^(30)^.

Similar perceptions were observed regarding access to diagnostic services for TB, including the RMT-TB, as well as important disparities within regions of the same country. A study conducted in Nepal on barriers to access identified that the main difficulties faced by the population are related to geographic barriers such as the long distance to services, poor road conditions, the availability of means of transport, and the costs associated with travel; in addition, the lack of health personnel trained in health units located in peripheral areas is frequently reported^(37)^.

The aforementioned issues indicate that, in addition to being geographically inaccessible, diagnostic facilities are constantly overburdened^(25)^. Therefore, geographic barriers produce a cascade effect on TB health indicators; for example, long distances to facilities hinder diagnosis, the notification rate, treatment initiation, and, consequently, its continuity^(38)^.

A systematic review indicated that low knowledge about TB among the population is the main aspect interfering with diagnosis and treatment, since most people do not have sufficient knowledge about what to do when they present certain symptoms and how to obtain appropriate care and treatment, especially in rural areas^(39)^. From the authors’ perspective, it is worth highlighting the socioeconomic factor as an important characteristic of disparity among populations; this reflects how TB is still a disease with a high social stigma.

Regional variations were also found in a study conducted in Ghana, where inadequate availability of diagnostic equipment/supplies in the country was observed, with significantly different proportions of diagnostic sites, with values ranging from 32,531 people per health facility in one region to 163,317 per facility in others^(38)^.

The presence of decentralized diagnostic services is necessary, since their lack directly affects the buildup of people affected by TB in the main centers, thereby preventing access to rapid diagnostic results^(39)^. Therefore, the resources arising from national TB programs must not be concentrated solely on acquiring RMT-TB machines; beyond that, management planning is needed so that these tools are available to the population in an accessible manner, aiming at equity in the provision of health services.

Furthermore, it is important to highlight that the incorporation of the RMT-TB may be limited by organizational factors and by the maintenance of previously established diagnostic practices, even in contexts with high technological capacity^(31)^. This result suggests that RMT-TB coverage does not depend solely on the availability of the test but also on the structure of the services, the care flows, and the training of the teams. Thus, challenges persist for the expansion of the use of the RMT-TB, especially in scenarios with high demand and regional heterogeneity.

The present study has limitations related to the methodology of the scoping review, such as the non-performance of a critical appraisal of the evidence reported in the included studies, which may limit the interpretation of the results, in addition to the heterogeneity of the different methodologies employed. Moreover, the results should be generalized with caution, since they addressed specific contexts that have distinct realities and health systems and different TB incidences.

The variation in coverage criteria among the studies indicates low conceptual standardization in the field and highlights the need for greater methodological and terminological clarity, especially for those that measure aspects of access to and coverage of the RMT-TB, even if in a secondary manner, so as to avoid the underidentification of relevant evidence.

The results of this review therefore present how coverage has been measured and reported in different studies and contexts, and which dimensions regarding the use of the test have been prioritized in these scenarios. Although these methodological differences limit direct comparability between countries, they provide an important overview of the local strategies for implementing the RMT-TB and of possible critical nodes in the TB care networks.

Therefore, this review provides an overview of RMT-TB coverage and identifies geographic, structural, and socioeconomic barriers that affect diagnosis; it highlights the need for conceptual standardization of coverage and reinforces the importance of the RMT-TB for early diagnosis, especially in populations in situations of vulnerability. The findings support the implementation of context-specific strategies, the training of teams, integration with traditional practices, and the promotion of equity, supporting evidence-based policies, improvement of care, and reduction of TB underreporting.

## CONCLUSION

The findings of this review revealed that, according to the analyzed literature, RMT-TB coverage has been operationalized and described in distinct ways in different countries and contexts, ranging from the percentage of people with TB initially tested with molecular methods to indicators of use in specific subgroups, equipment availability, and geographic accessibility.

The studies indicate important advances in the incorporation of the RMT-TB as a diagnostic method, especially in high-TB-burden countries, although in many scenarios its use remains below the international targets set by the WHO. The results suggest that low coverage is strongly associated with structural and socioeconomic barriers, dependence on external funding, the absence of clear national guidelines for the implementation of molecular tests, and insufficient decentralization of diagnostic services.

For the RMT-TB to reach its full diagnostic potential and have a positive impact on TB control, a renewed commitment from national governments to the decentralization of services, the availability and training of professionals, and the development of local and regional policies is necessary. Strengthening funding, eliminating access barriers, and adopting a more ambitious approach to the sustained expansion of coverage are essential steps to reduce inequities and advance in the effective diagnosis of TB. Special attention should be given to populations in situations of vulnerability, prioritizing strategies that reduce stigma, expand early diagnosis, and decrease underreporting. In the research field, the adoption of standardized criteria aligned with the guidelines of the WHO to measure RMT-TB coverage is recommended, enabling comparisons between countries and the continuous monitoring of the established targets.

## Data Availability

The entire dataset supporting the results of this study was published within the article itself.
