# Supplementary material for: Operationalization of rapid molecular test coverage for tuberculosis in different international contexts: a scoping review
Source: Rev Esc Enferm USP. 2026 Aug 10;60:e20260172. doi: 10.1590/1980-220X-REEUSP-2026-0172en (PMC13456686; doi:10.1590/1980-220X-REEUSP-2026-0172en)
Supplement: Translation

# Translation: Operacionalização da cobertura do teste rápido molecular para tuberculose em diferentes contextos internacionais: revisão de escopo

**DOI:** 10.1590/1980-220X-REEUSP-2026-0172pt

**Authors:** Diogo Henrique Mendes da Silva, Ana Beatriz Marques Valença, Melisane Regina Lima Ferreira, Mariana Gaspar Botelho Funari de Faria, Rubia Laine de Paula Andrade, Rebeca Sousa Braga, Aline Aparecida Monroe

**Affiliations:** 1 Universidade de São Paulo, Escola de Enfermagem de Ribeirão Preto, Programa de Pós-Graduação Enfermagem em Saúde Pública, Ribeirão Preto, SP, Brasil.; 2 Ministério da Saúde, Secretaria de Vigilância em Saúde e Ambiente, Coordenação-Geral de Vigilância da Tuberculose, Micoses Endêmicas e Micobactérias Não Tuberculosas, Brasília, DF, Brasil.; 3 Universidade de São Paulo, Escola de Enfermagem de Ribeirão Preto, Departamento de Enfermagem Materno-Infantil e Saúde Pública, Ribeirão Preto, SP, Brasil.

**Keywords:** Tuberculose, Técnicas de Diagnóstico Molecular, Testes de Diagnóstico Rápido.

## Abstract

**Objetivo:**

Mapear estudos sobre a cobertura do teste rápido molecular para tuberculose em diferentes contextos internacionais.

**Método:**

Revisão de escopo, conduzida segundo as recomendações do PRISMA-ScR, com buscas nas bases de dados da MEDLINE, Scopus, Web of Science, Embase, LILACS e BDTD, abrangendo o período de 2011 a 2025.

**Resultados:**

Os 20 estudos incluídos evidenciaram heterogeneidade conceitual na operacionalização da cobertura, abrangendo desde proporções de testagem em grupos populacionais específicos até indicadores de disponibilidade de equipamentos e acessibilidade geográfica, sem padronização entre países e contextos. Os valores variaram de menos de 1% a aproximadamente 80%, com diferenças expressivas entre países, populações-alvo e períodos analisados. Desafios socioeconômicos,estruturais, financeiros e de acesso às instalações dificultam a utilização do teste.

**Conclusão:**

A cobertura do teste varia entre países e contextos, limitada por barreiras estruturais, financeiras e pela ausência de padronização conceitual e metodológica na sua mensuração. Ampliar e monitorar a cobertura de forma comparável exige compromisso governamental, descentralização e adoção de critérios uniformes alinhados às diretrizes da Organização Mundial da Saúde.

## INTRODUÇÃO

Embora curável, a tuberculose (TB) ainda constitui um desafio para os sistemas de saúde em todo o mundo. O alto número de pessoas que adoecem em países em desenvolvimento reforça a necessidade da continuidade das estratégias de prevenção e controle^(1)^. Em 2025, estima-se que mais de 10 milhões de pessoas continuem adoecendo por TB todos os anos e mais de um milhão morrem em decorrência da doença^(2)^.

A baciloscopia, embora seja uma técnica de diagnóstico simples e de baixo custo, apresenta limitações, como a baixa sensibilidade e especificidade^(3,4)^. A princípio, essas limitações eram superadas pela realização de culturas microbiológicas, consideradas como o padrão de referência para o diagnóstico da TB, TB drogarresistente (TB-DR) e micobactérias não tuberculosas^(3)^. No entanto, a principal desvantagem da cultura é o tempo prolongado para obtenção dos resultados, normalmente entre quatro e oito semanas^(5)^.

Devido a essas limitações, foram desenvolvidos novos métodos diagnósticos para aprimorar a detecção da doença e progredir rumo ao alcance dos Objetivos de Desenvolvimento Sustentável (ODS), adotados em setembro de 2015, relacionados à resposta social e de saúde para o combate à TB^(6)^.

Nesse sentido, dentre os testes moleculares desenvolvidos, a tecnologia GeneXpert MTB/RIF, conhecida no Brasil como Teste Rápido Molecular para Tuberculose (TRM-TB), foi recomendada mundialmente pela Organização Mundial da Saúde (OMS) a partir de dezembro de 2010^(5)^, a qual posteriormente foi aprimorada e substituída pelo Xpert MTB/RIF Ultra em 2019, uma versão com maior sensibilidade e especificidade^(7)^. Ambos os testes têm a capacidade de detectar o complexo *Mycobacterium tuberculosis* e identificar cepas resistentes à rifampicina em amostras biológicas em aproximadamente duas horas^(3,4,7)^.

Após a recomendação, vários países passaram a adotar a tecnologia no diagnóstico de novos adoecimentos por TB. No entanto, estudos realizados em condições de rotina assistencial mostram que o TRM-TB aumentou a detecção de casos e melhorou o acesso ao diagnóstico^(5,8)^.

Apesar das recomendações da implementação do teste em diferentes contextos, persistem lacunas não apenas quanto à sua aplicação prática nos sistemas de saúde, mas sobretudo quanto à forma como sua cobertura é definida e mensurada. A ausência de critérios padronizados para operacionalizar a cobertura do TRM-TB dificulta a comparação entre países e a avaliação do alcance das metas estabelecidas pela OMS, configurando uma lacuna científica que esta revisão se propõe a mapear.

Para compreender essa lacuna, cobertura, acesso, utilização e disponibilidade são conceitos distintos, embora frequentemente utilizados de forma sobreposta na literatura. A cobertura, em sentido ampliado, expressa o grau de interação entre o serviço e a população a que se destina, desde a alocação de recursos até a obtenção do desfecho pretendido^(9)^.

Diante disso, este estudo tem como objetivo mapear estudos sobre a cobertura do TRM-TB em diferentes contextos internacionais, identificando como esse indicador tem sido operacionalizado e descrito na literatura, de modo a subsidiar estratégias de gestão e políticas de saúde voltadas ao controle da TB.

## MÉTODO

### Tipo de Estudo

Trata-se de uma revisão de escopo, realizada a partir das recomendações do *checklist Preferred Reporting Items for Systematic reviews and Meta-Analyses extension for Scoping Reviews* (PRISMA-ScR)^(10)^, para orientar o relato dos resultados. A condução seguiu o manual JBI^(11)^, que estabelece as etapas metodológicas da revisão de escopo: 1) definição da questão de pesquisa; 2) levantamento dos estudos relevantes; 3) triagem e avaliação preliminar dos estudos; 4) análise das informações dos estudos; e 5) organização, síntese e apresentação dos resultados. O PRISMA-ScR foi utilizado como guia de transparência no relato de cada uma dessas etapas. O protocolo desta revisão foi registrado na base *Open Science Framework* (OSF) sob o DOI: doi: https://doi.org/10.17605/OSF.IO/6EUH7.

### Coleta de Dados

A pergunta norteadora “Como a cobertura do TRM-TB para o diagnóstico de pessoas com TB ou com sinais e sintomas sugestivos de TB tem sido operacionalizada e descrita em diferentes contextos internacionais?” foi desenvolvida com base na metodologia mnemônica População, Conceito e Contexto (PCC). Nesse modelo, a População (P) foi representada pelas pessoas com TB ou com sinais e sintomas sugestivos de TB; o Conceito (C) correspondeu à cobertura do TRM-TB; e o Contexto (C) abrangeu os diferentes contextos internacionais.

Com base nessa questão, este estudo considerou a concepção ampliada de cobertura de serviços de saúde proposta por Tanahashi, entendida como o grau de interação entre o serviço e a população a que se destina, abrangendo desde a disponibilidade e alocação de recursos até a obtenção do desfecho pretendido^(9)^. Pensando nas dimensões de disponibilidade, acessibilidade, aceitabilidade, contato e efetividade, foram considerados como indicadores de cobertura a proporção de testes realizados em grupos populacionais, a disponibilidade de equipamentos nos serviços de saúde e a acessibilidade geográfica às instalações diagnósticas, conforme a operacionalização apresentada em cada estudo. Tais dimensões consideradas como indicadores de cobertura foram identificadas a partir da leitura dos estudos incluídos.

Para o levantamento dos estudos relevantes, foi realizada uma busca eletrônica nas seguintes bases de dados: *Scopus, Web of Science, Medical Literature Analysis and Retrieval System Online* (MEDLINE), Literatura Latino-Americana e do Caribe em Ciências da Saúde (LILACS) e *Embase*. Também foram realizadas buscas para identificar a literatura cinzenta junto à Biblioteca Digital Brasileira de Teses e Dissertações (BDTD).

A busca eletrônica ocorreu em dezembro de 2025 empregando palavras-chave identificadas previamente na literatura, e descritores indexados à plataforma de Descritores em Ciências da Saúde (DeCS), bem como seus sinônimos em português e espanhol. Também foram utilizados descritores indexados à plataforma *The Medical Subject Heading* (MeSH) para a elaboração das estratégias de busca na língua inglesa. Para relacionar os descritores e palavras-chave entre si, foram aplicados os operadores booleanos *AND* e *OR.* Os descritores, palavras-chave, período e as estratégias de busca realizadas para cada base de dados são apresentadas no Quadro 1. A estratégia de busca foi validada pelos coautores, integrantes do mesmo grupo de pesquisa. Os filtros de idioma (inglês, português e espanhol) foram aplicados na etapa de triagem dos estudos.

**Quadro 1:** Estratégias utilizadas para o levantamento de estudos aplicadas a cada base de dados – Ribeirão Preto, SP, Brasil, 2025.

Bases de dados	Estratégias de busca
MEDLINE	((“tuberculosis” [MeSH Terms] OR “tuberculosis” [All Fields] OR “tuberculoses” [All Fields] OR “tb” [All Fields]) AND (“xpert” [All Fields] OR “genexpert” [All Fields] OR “gene xpert” [All Fields] OR “cepheid” [All Fields] OR “cepheids” [All Fields] OR “rapid molecular test” [All Fields]) AND (“coverage” [All Fields] OR “coverages” [All Fields] OR “diagnostic coverage” [All Fields])) AND (medline[Filter]) AND (2011:2025[pdat])
Scopus	(TITLE-ABS-KEY (tuberculosis OR tb) AND TITLE-ABS-KEY (xpert OR genexpert OR “gene xpert” OR cepheid OR “rapid molecular test” ) AND TITLE-ABS-KEY (coverage OR “diagnostic coverage”) ) AND PUBYEAR > 2010 AND PUBYEAR < 2026
Web of Science	tuberculosis OR tb (Topic) and xpert OR genexpert OR “gene xpert” OR cepheid OR “rapid molecular test” (Topic) and coverage OR “diagnostic coverage” (Topic) and 2025 or 2024 or 2023 or 2022 or 2021 or 2020 or 2019 or 2018 or 2017 or 2016 or 2015 or 2014 or 2013 or 2012 or 2011 (Publication Years)
Embase	#1 (‘tuberculosis’/exp OR tuberculosis OR ‘tb’/exp OR tb) AND [2011-2025]/py #2 xpert OR genexpert OR ‘gene xpert’ OR cepheid OR ‘rapid molecular test’ #3 coverage OR ‘diagnostic coverage’ #4 #1 AND #2 AND #3
LILACS	(tuberculosis OR tuberculose OR tb) AND (xpert OR genexpert OR “gene xpert” OR cepheid OR “rapid molecular test” OR “teste rápido molecular” OR trm-tb OR “teste molecular rápido” OR “prueba rápida molecular” OR prm OR “prueba molecular rápida”) AND (coverage OR “diagnostic coverage” OR cobertura OR “cobertura diagnóstica” OR “cobertura de diagnóstico”) AND (year_cluster:[2011 TO 2025])
Biblioteca Digital Brasileira de Teses e Dissertações	“(Todos os campos:tuberculosis OR tuberculose OR tb E Todos os campos:xpert OR genexpert OR “gene xpert” OR cepheid OR “rapid molecular test” OR “teste rápido molecular” OR trm-tb OR “teste molecular rápido” OR “prueba rápida molecular” OR prm OR “prueba molecular rápida” E Todos os campos:coverage OR “diagnostic coverage” OR cobertura OR “cobertura diagnóstica” OR “cobertura de diagnóstico”)”

LILACS: Literatura Latino-Americana e do Caribe em Ciências da Saúde.

### Critérios de Seleção

Os critérios de elegibilidade para esta revisão de escopo englobaram estudos que abordaram a cobertura ou percentual de utilização do TRM-TB (GeneXpert MTB/RIF e/ou Xpert MTB/RIF Ultra), entendidos como indicadores quantitativos de uso (cobertura com base na utilização do TRM-TB entre as pessoas com sinais e sintomas sugestivos de TB; cobertura com base na utilização do TRM-TB entre as pessoas diagnosticadas com TB; cobertura com base na utilização do TRM-TB em pessoas com TB-DR; cobertura com base na utilização do TRM-TB em pessoas vivendo com HIV; e cobertura com base na utilização do TRM-TB entre as pessoas que testaram negativo na baciloscopia de escarro) e/ou disponibilidade ou acesso ao teste (cobertura com base no número de máquinas de TRM-TB em relação ao número de unidades de saúde; e cobertura do TRM-TB com base na distância da população às instalações de diagnóstico). Assim, foram incluídos estudos que apresentavam esses indicadores como objetivo principal ou como achado secundário, desde que os dados quantitativos estivessem claramente descritos; que tiveram na população analisada pessoas com TB ou com sinais e sintomas sugestivos de TB, independentemente da forma clínica ou condições de vulnerabilidade; e foram publicados entre 2011 e 2025.

A opção por considerar esses estudos decorre de sua ampla difusão em programas nacionais e internacionais de controle da TB, de seu reconhecimento pela OMS como referência para o diagnóstico molecular rápido e da maior disponibilidade de estudos que adotam métricas comparáveis. Essa delimitação buscou reduzir a heterogeneidade entre tecnologias distintas e permitir maior consistência na análise da cobertura. A escolha do período baseia-se na recomendação adotada pela OMS para a utilização do TRM-TB na testagem da TB e da TB-DR, a partir de dezembro de 2010^(2)^.

Foram elegíveis estudos publicados em inglês, português e espanhol, primários ou secundários, revisados ou não por pares, desde que em seu escopo estivesse contemplado algum indicador de cobertura do TRM-TB em qualquer zona geográfica do mundo, utilizando qualquer abordagem e desenho metodológico. Foram consideradas teses, dissertações, trabalhos de conclusão de curso e resumos de conferências.

Foram excluídos protocolos, manuais, diretrizes, notas técnicas, cartas, editoriais e comentários, por não apresentarem dados empíricos quantitativos acerca da utilização ou cobertura do teste foco desta revisão. Ademais, estudos que não apresentassem em seu título e resumo informações claras relacionadas à cobertura ou ao TRM-TB foram descartados ainda na etapa de triagem de títulos e resumos. Também foram excluídos estudos que agregam outros tipos de testes de diagnósticos moleculares distintos do GeneXpert MTB/RIF ou Xpert MTB/RIF Ultra, por envolverem tipos de amostra e procedimentos laboratoriais não comparáveis ao TRM-TB definido para esta revisão.

### Extração e Análise dos Dados

Após o processo de busca nas bases de dados, as publicações foram exportadas para a plataforma *Rayyan AI*, uma ferramenta de auxílio às pesquisas de revisão para identificar e eliminar duplicatas e selecionar os estudos elegíveis^(12)^.

A extração foi realizada em duas partes: a primeira foi conduzida por dois revisores de maneira independente, que avaliaram os títulos e resumos das referências identificadas, selecionando estudos potencialmente elegíveis com base na pergunta norteadora e critérios de elegibilidade. Divergências foram solucionadas por um terceiro autor habilitado. A segunda parte consistiu na leitura e avaliação na íntegra dos estudos pré-selecionados, documentando-se as razões de exclusão em fluxograma baseado no PRISMA-ScR^(10)^.

As informações dos estudos incluídos foram extraídas a partir de um instrumento adaptado para utilização em planilha do Excel contendo as seguintes variáveis: autor(es); título; periódico; tipo de publicação; idioma do estudo; país onde foi realizado o estudo; ano de publicação; objetivo; delineamento do estudo; período do estudo; população do estudo; tamanho da amostra; definição operacional de cobertura do TRM-TB; principais resultados relacionados à cobertura e/ou percentual de utilização do teste; população a qual se destina o teste; e principais discussões e conclusões dos autores.

Dessas variáveis, foram apresentadas aquelas diretamente relacionadas à pergunta de pesquisa: definição operacional da cobertura, valores de cobertura mensurados, delineamento do estudo e país de realização. As demais variáveis foram utilizadas para orientar a síntese narrativa e a construção das categorias analíticas.

Todos os resultados foram apresentados em formato de um quadro descritivo contendo a definição operacional da cobertura utilizada e os valores de cobertura mensurados nos estudos. Além disso, foram elencadas duas categorias de análise para compor a discussão dos resultados: fatores associados à baixa cobertura e acesso ao diagnóstico pelo TRM-TB. Essas categorias emergiram durante a leitura e análise dos estudos incluídos, a partir dos temas recorrentes identificados nas suas discussões e conclusões.

### Aspectos Éticos

Esta revisão de escopo utilizou exclusivamente fontes de literatura secundária disponíveis publicamente, sem coleta de dados primários ou informações pessoais identificáveis. Por essa razão, e em conformidade com as normas éticas aplicáveis, não foi necessária a aprovação de um Comitê de Ética em Pesquisa nem a obtenção de Termo de Consentimento Livre e Esclarecido.

## RESULTADOS

Os estudos incluídos foram analisados com foco na forma como a cobertura do TRM-TB foi operacionalizada e descrita em cada contexto. Foram identificados 502 registros nas bases de dados. Após a remoção dos estudos duplicados, 220 foram submetidos à leitura de títulos e resumos. Realizada a leitura, a presente revisão incluiu 20 estudos que se enquadraram nos critérios de inclusão e responderam à pergunta de pesquisa. As razões de exclusão para artigos lidos na íntegra foram documentadas conforme demonstrado no fluxograma da Figura 1.

**Figura 1:** Fluxograma de inclusão e exclusão das publicações de acordo com as recomendações do PRISMA-ScR – Ribeirão Preto, Brasil, 2025. LILACS: Literatura Latino-Americana e do Caribe em Ciências da Saúde; TRM-TB: Teste Rápido Molecular para a Tuberculose.

O período de publicação dos estudos incluídos foi de 2017 a 2025^(13,14,15,16,17,18,19,20,21,22,23,24,25,26,27,28,29,30,31,32)^, com maior concentração em 2020^(19,20,21)^, 2021^(22,23,24)^ e 2024^(28,29,30)^, anos em que foram identificadas três publicações cada. Com relação ao idioma de publicação, 19 estudos estavam disponíveis em inglês^(13,14,15,16,17,18,19,21,22,23,24,25,26,27,27,28,29,30,31,32)^ e um em espanhol^(20)^. No que se refere ao delineamento dos estudos, observou-se uma predominância de abordagens quantitativas, com apenas um estudo envolvendo métodos mistos^(22)^. Em sua maiorparte, foram estudos de coorte^(16,17,19,24)^ (Quadro 2). Entre os estudos incluídos, foram identificadas sete formas distintas de operacionalizar a cobertura do TRM-TB, categorizados a partir das informações disponíveis, a saber: 1) cobertura com base na utilização do TRM-TB entre as pessoas com sinais e sintomas sugestivos de TB (n = 8)^(15,17,18,24,26,27,29,30)^; 2) cobertura com base na utilização do TRM-TB entre as pessoas diagnosticadas com TB (n = 4)^(13,14,19,20)^; 3) cobertura com base na utilização do TRM-TB em pessoas com TB-DR (n = 2)^(16,31)^; 4) cobertura com base na utilização do TRM-TB em pessoas vivendo com HIV (n = 2)^(28,32)^; 5) cobertura com base na utilização do TRM-TB entre as pessoas que testaram negativo na baciloscopia de escarro (n = 2)^(21,23)^; 6) cobertura com base no número de máquinas de TRM-TB em relação ao número de unidades de saúde (n = 1)^(22)^; e 7) cobertura do TRM-TB com base na distância da população às instalações de diagnóstico (n = 1)^(25)^ (Quadro 2).

**Quadro 2:** Apresentação dos estudos incluídos na revisão – Ribeirão Preto, SP, Brasil, 2025.

Autor, ano e país	Tipo de publicação	Delineamento do estudo	Objetivos do estudo	Definição operacional da cobertura	Valores de cobertura do Teste Rápido Molecular
Rees et al., 2017^(13)^; África do Sul	Artigo	Estudo retrospectivo	Avaliar as tendências diagnósticas em pacientes tratados para TB em um subdistrito rural da África do Sul, em relação à implementação do TRM-TB e seu algoritmo acompanhante, utilizando dados de rotina.	Cobertura com base na utilização do TRM-TB entre as pessoas diagnosticadas com TB.	De 0% em 2010 para 69,9% em 2015 no subdistrito de Greater Tzaneen, África do Sul.
Zemlyansky et al., 2017^(14)^; Rússia	Artigo	Estudo transversal	Avaliar a eficácia e desenvolver um modelo ótimo para o diagnóstico laboratorial de tuberculose usando TRM-TB.	Cobertura com base na utilização do TRM-TB entre as pessoas diagnosticadas com TB.	75,3% entre novos casos e 80,8% em recidivas na região de Belgorod, Rússia em 2015.
Awan et al., 2018^(15)^; Paquistão	Artigo	Estudo transversal	Preencher as lacunas na literatura sobre as potenciais restrições na implementação do TRM-TB em ambientes programáticos com uma alta carga de TB-DR.	Cobertura com base na utilização do TRM-TB entre as pessoas com sinais e sintomas sugestivos de TB.	44,3% entre julho de 2013 e junho de 2015 em Karachi, Paquistão.
Evans et al., 2018^(16)^; África do Sul	Artigo	Estudo de coorte retrospectiva	Analisar o impacto da introdução do TRM-TB e da descentralização do tratamento na ligação ao cuidado e nos resultados do tratamento da TB-DR em Joanesburgo, África do Sul.	Cobertura com base na utilização do TRM-TB em pessoas com TB-DR.	43,4% entre julho de 2011 e junho de 2012 e 60,5% entre julho de 2013 a junho de 2014, em Joanesburgo, África do Sul.
Farr et al., 2019^(17)^; Uganda	Artigo	Estudo de coorte prospectiva	Caracterizar até que ponto os pacientes de TB em Uganda estavam recebendo cuidados de acordo com as diretrizes nacionais e internacionais.	Cobertura com base na utilização do TRM-TB entre as pessoas com sinais e sintomas sugestivos de TB.	19,5% em 2017 em 24 centros de saúde na Uganda.
Kabasakalyan et al., 2019^(18)^; Armênia	Artigo	Estudo observacional retrospectivo	Avaliar a mudança no perfil diagnóstico laboratorial do *Mycobacterium tuberculosis* após a introdução do TRM-TB.	Cobertura com base na utilização do TRM-TB entre as pessoas com sinais e sintomas sugestivos de TB.	58% no período de 2013 a 2017 na Armênia.
Wu et al., 2020^(19)^; China	Artigo	Estudo de coorte retrospectiva	Avaliar o efeito da implementação do TRM-TB na detecção de casos de TB pulmonar.	Cobertura com base na utilização do TRM-TB entre as pessoas diagnosticadas com TB.	54% no primeiro semestre de 2018 em Xangai, China.
Agredo and Osorio, 2020^(20)^; Colômbia	Artigo	Estudo descritivo	Descrever a cobertura e a fidelidade no uso do TRM-TB em pacientes com tuberculose pulmonar em uma cidade com alta carga da doença na Colômbia.	Cobertura com base na utilização do TRM-TB entre as pessoas diagnosticadas com TB.	10,35% entre 2013 e 2019 no município de Cali, Colômbia.
Creswell et al., 2020^(21)^; Guatemala	Artigo	Estudo de avaliação programática	Avaliar o impacto da introdução do TRM-TB como um teste de acompanhamento após a microscopia de esfregaço no número total de notificações de TB pulmonar.	Cobertura com base na utilização do TRM-TB entre as pessoas que testaram negativo na baciloscopia de escarro.	39,6% entre abril de 2015 e março de 2016 na Guatemala.
Ntinginya et al., 2021^(22)^; Uganda, Quênia e Tanzânia	Artigo	Quantitativo e Qualitativo	Investigar as barreiras e oportunidades para maximizar a captação e utilização de diagnósticos moleculares em ambientes de rotina de assistência médica.	Cobertura com base no número de máquinas de TRM-TB em relação ao número de unidades de saúde.	De setembro de 2016 a dezembro de 2017, 74% na Uganda, 39% na Tanzânia e 42% no Quênia.
Li et al., 2021^(23)^; China	Artigo	Estudo transversal de avaliação antes e depois	Avaliar o impacto do Projeto de Tuberculose China-Gates Fase III no acesso a diagnósticos e tratamentos de qualidade para TB.	Cobertura com base na utilização do TRM-TB entre as pessoas que testaram negativo na baciloscopia de escarro.	0% em 2015, 16% em 2018 utilizando apenas o TRM e 34,4% em 2018 utilizando TRM-TB + cultura, em 12 localidades de três províncias da China: Zhejiang, Jilin e Ningxia.
Turaev et al., 2021^(24)^; Uzbequistão	Artigo	Estudo de coorte prospectiva	Avaliar a cobertura do TRM-TB entre pessoas com TB presuntiva, fatores associados a ele e os atrasos envolvidos.	Cobertura com base na utilização do TRM-TB entre as pessoas com sinais e sintomas sugestivos de TB.	24% em 2018 e 46% em 2019 no Uzbequistão.
Milaham et al., 2022^(25)^; Nigéria	Artigo	Estudo ecológico retrospectivo	Investigar a distribuição espacial das taxas de notificação de casos de TB, diagnósticos e cobertura entre as Áreas de Governo Local.	Cobertura do TRM-TB com base na distância da população às instalações de diagnóstico.	49% da população vivia a uma distância maior que 20km de uma instalação de TRM-TB entre 2017 e 2019 no estado de Katsina, Nigéria.
Ishengoma et al., 2023^(26)^; Tanzânia	Resumo publicado em anais de evento	Estudo descritivo	Avaliar a eficácia das clínicas móveis na Tanzânia como uma estratégia para detectar casos de TB não diagnosticados em áreas de difícil acesso.	Cobertura com base na utilização do TRM-TB entre as pessoas com sinais e sintomas sugestivos de TB.	34,5% em 8 regiões da Tanzânia utilizando clínicas móveis.
Getahun et al., 2023^(27)^; Etiópia	Artigo	Estudo retrospectivo	Revisar os registros disponíveis em unidades de saúde selecionadas em Addis Ababa, Etiópia, e avaliar os impactos do TRM-TB no número de casos de TB detectados e nos sucessos nos resultados do tratamento.	Cobertura com base na utilização do TRM-TB entre as pessoas com sinais e sintomas sugestivos de TB.	71,1% entre 2015 e 2018 em Addis Ababa, Etiópia.
Faria et al., 2024^(28)^; Brasil	Artigo	Estudo ecológico	Analisar o diagnóstico de tuberculose pulmonar não confirmada antes e depois da implementação do TRM-TB para pessoas vivendo com HIV em municípios do estado de São Paulo, Brasil.	Cobertura com base na utilização do TRM-TB em pessoas vivendo com HIV	49,4% entre 2010 e 2020 no estado de São Paulo, Brasil.
Pank et al., 2024^(29)^; Papuá-Nova Guiné	Artigo	Estudo de implementação prospectivo	Avaliar a implementação inicial do *Systematic Island-Wide Engagement & Elimination Project for TB* em Daru usando uma iniciativa de Melhoria Contínua da Qualidade.	Cobertura com base na utilização do TRM-TB entre as pessoas com sinais e sintomas sugestivos de TB.	77,9% entre maio e dezembro de 2023 em Daru, Papuá-Nova Guiné.
Dakulala et al., 2024^(30)^; Papuá-Nova Guiné	Artigo	Estudo retrospectivo	Rastrear a população da ilha Daru usando uma van móvel equipada com máquinas de TRM-TB e raio-X digital com software de diagnóstico assistido por computador para TB.	Cobertura com base na utilização do TRM-TB entre as pessoas com sinais e sintomas sugestivos de TB.	79,7% entre fevereiro a outubro de 2018 e fevereiro a março de 2019, em Daru, Papuá-Nova Guiné usando uma van móvel.
Mok et al., 2025^(31)^; Coreia do Sul	Artigo	Estudo de coorte retrospectiva	Avaliar a cobertura do teste molecular de suscetibilidade a medicamentos entre pacientes com TB-DR pulmonar na Coreia do Sul e identificar fatores que influenciam a sua falta de implementação.	Cobertura com base na utilização do TRM-TB em pessoas com TB-DR.	22,1% no total de pacientes, sendo 23,5% nos novos casos e 19,9% em pacientes previamente tratados na Coreia do Sul.
Motlhaoleng et al., 2025^(32)^; África do Sul	Artigo	Estudo descritivo	Avaliar a cascata de cuidados do *Targeted Universal Testing for TB* para descrever sua implementação dentro da continuidade de cuidados para as pessoas vivendo com HIV em instalações de saúde de alto volume (≥6500 pacientes), médio volume (3500 a <6500 pacientes) e baixo volume (<3500 pacientes)	Cobertura com base na utilização do TRM-TB em pessoas vivendo com HIV	No distrito rural de Zululand, as instalações de alto volume apresentaram 44,4% em 2022 e 43,3% em 2023; as de médio volume, 43,1% e 32,0%; e as de baixo volume, 55,0% e 63,9%, respectivamente. No Município de eThekwini (distrito urbano), as instalações de alto volume registraram 22,3% em 2022 e 28,3% em 2023; as de médio volume, 20,5% e 17,1%; e as de baixo volume, 2,2% e 5,5%.

TB: Tuberculose; TRM-TB: Teste Rápido Molecular para Tuberculose; TB-DR: Tuberculose Drogarresistente; HIV: *Human Immunodeficiency Virus*.

Em termos de operacionalização, as categorias diferem quanto ao numerador, denominador e população-alvo considerados. Nas categorias baseadas na utilização do teste, o numerador correspondeu ao número de pessoas testadas com TRM-TB, e o denominador variou conforme o grupo populacional: pessoas com sinais e sintomas sugestivos, pessoas diagnosticadas com TB, pessoas com TB-DR, pessoas vivendo com HIV e pessoas com baciloscopia negativa. Na categoria baseada na disponibilidade de equipamentos, o numerador correspondeu ao número de máquinas instaladas e o denominador ao número de unidades de saúde. Na categoria baseada na acessibilidade geográfica, o numerador correspondeu à população residente a mais de determinada distância de uma instalação diagnóstica e o denominador à população total da área estudada.

Quanto ao número de estudos por países onde foram desenvolvidos: África do Sul (n = 3)^(13,16,32)^, China (n = 2)^(19,23)^, Papuá-Nova Guiné (n = 2)^(29,30)^; Coreia do Sul (n = 1)^(31)^, Armênia (n = 1)^(18)^, Uzbequistão (n = 1)^(24)^, Rússia (n = 1)^(14)^, Paquistão (n = 1)^(15)^, Etiópia (n = 1)^(27)^, Uganda (n = 1)^(17)^, Tanzânia (n = 1)^(26)^, Nigéria (n = 1)^(25)^, Brasil (n = 1)^(28)^, Colômbia (n = 1)^(20)^ e Guatemala (n = 1)^(21)^. Houve ainda um estudo envolvendo três países da África: Uganda, Quênia e Tanzânia (n = 1)^(22)^.

A partir da leitura dos estudos incluídos, emergiram duas categorias analíticas para organizar os principais achados: fatores associados à baixa cobertura e acesso ao diagnóstico pelo TRM-TB.

### Fatores Associados à Baixa Cobertura

Nos estudos que reportaram baixa cobertura do TRM-TB, foram identificadas barreiras à utilização do teste, incluindo fatores socioeconômicos e culturais e limitações estruturais dos sistemas de saúde. Entre estas, destacaram-se: baixa busca ativa, qualidade insuficiente das amostras, uso de equipamentos restrito a um turno de trabalho, erros no preenchimento dos pedidos, subfinanciamento da saúde, dificuldades de gestão entre gestores e profissionais, escassez de recursos humanos qualificados e ausência de infraestrutura básica, como abastecimento de água e eletricidade^(22,24,28,29,32)^.

Alguns estudos relataram o uso de máquinas de TRM-TB com apenas quatro módulos, associado a um menor número de pessoas testadas e à menor detecção, especialmente de TB-DR; nesses contextos, foram descritas experiências com equipamentos de 16 módulos para ampliar a capacidade de testagem^(25,26)^.

Também foi relatada, em determinados países, a dependência de orçamentos de saúde financiados por doadores externos, com relatos de que oscilações econômicas nesses países afetaram a continuidade da oferta de diagnóstico e tratamento^(22)^. Além disso, um estudo mencionou dificuldades dos programas de TB em planejar intervenções e investimentos com base em estimativas pontuais de carga de TB da OMS, sujeitas a variações anuais e amplos intervalos de confiança^(21)^.

### Acesso ao Diagnóstico Pelo TRM-TB

Alguns estudos descreveram limitações de acesso da população às unidades que ofertam diagnóstico por TRM-TB. No estado de Katsina, na Nigéria, a distribuição das instalações foi considerada desigual, com 100% da população de duas Áreas de Governo Local (LGAs) e 75% de oito LGAs localizadas a mais de 20km de qualquer ponto de diagnóstico, indicando maior distância a ser percorrida pelas pessoas residentes nessas áreas^(25)^. Além disso, ressaltou-se a dificuldade de infraestrutura e assistência médica em áreas rurais quando comparadas às áreas urbanas^(32)^.

Em Uganda, Tanzânia e Quênia, o TRM-TB não era utilizado de forma universal para todas as pessoas com sinais e sintomas sugestivos de TB; sua indicação era prioritária para pessoas vivendo com HIV ou com contato prévio com a TB-DR. A partir de 2019, a recomendação nesses países passou a abranger todas as pessoas com sinais e sintomas sugestivos de TB^(22)^.

Na coorte realizada em Tashkent e Bukhara, no Uzbequistão, observaram-se diferenças regionais no acesso ao teste. A proporção de pessoas que não realizaram TRM-TB foi maior em Bukhara (73%; IC95%: 69–76) do que em Tashkent (28%; IC95%: 25–30) (p < 0,001). Em Bukhara, 51% das pessoas com TB procuraram unidades sem serviço de diagnóstico, em comparação a 9% em Tashkent; além disso, 80% dos residentes de Bukhara acessaram unidades rurais periféricas, enquanto 88% dos residentes de Tashkent estavam vinculados a unidades localizadas em áreas centrais^(24)^.

O estudo realizado na África do Sul aponta fragilidades quanto ao uso do TRM-TB entre pessoas vivendo com HIV nos distritos de Zululand (rural) e eThekwini (urbano), ao se considerar instalações de alto volume (≥6500 pacientes), médio volume (3500 a <6500 pacientes) e baixo volume (<3500 pacientes). A situação mais crítica acontece no contexto urbano, especialmente nas instalações de médio e baixo volume^(32)^.

Em doze localidades de três províncias da China, entre 2015 e 2018, foi observado aumento do acesso a diagnósticos de qualidade para TB, relacionado ao incremento do uso do TRM-TB e à expansão dos serviços de diagnóstico para pessoas com baciloscopia negativa^(23)^.

## DISCUSSÃO

A partir do levantamento das publicações, verificou-se maior número de estudos realizados no continente africano e asiático, o que pode estar relacionado ao elevado número e incidência da TB nesses locais. Geograficamente, a maioria das pessoas que desenvolveram TB nos últimos anos estavam nas regiões do Sudeste Asiático (45%), África (24%) e Pacífico Ocidental (17%), com proporções menores no Mediterrâneo Oriental (8,6%), nas Américas (3,2%) e na Europa (2,1%)^(2)^.

Um achado central desta revisão foi a heterogeneidade conceitual e operacional do termo “cobertura” associado ao TRM-TB. Embora a OMS utilize como critério base a proporção de pessoas com TB inicialmente testadas com um teste molecular rápido^(2)^, apenas quatro estudos incluídos adotaram essa definição de forma explícita^(13,14,19,20)^. Observou-se, contudo, que outras pesquisas avaliaram aspectos relacionados à cobertura do teste, como disponibilidade de equipamentos, proporção de testagem em subgrupos populacionais ou acesso ao diagnóstico, sem nomeá-los como medidas de cobertura.

A diversidade observada nos estudos reflete a amplitude do próprio conceito de cobertura de serviços de saúde que, em sentido ampliado, abrange desde a disponibilidade de recursos até a obtenção do desfecho pretendido^(9)^, aproximando-se em alguns estudos de conceitos como acesso e utilização.

Essa imprecisão conceitual, embora não impacte operacionalizações legítimas, tem implicações diretas para a comparabilidade dos dados: quando alguns estudos mensuram cobertura como proporção de pessoas testadas entre sintomáticos respiratórios, outros como disponibilidade de equipamentos e outros como acessibilidade geográfica, os resultados tornam-se incomparáveis entre si. Os diferentes resultados refletem dimensões distintas do mesmo fenômeno, e equipará-los dificulta tanto o diagnóstico da situação quanto a avaliação do impacto das intervenções. A ausência de um marco conceitual comum para operacionalizar a cobertura do TRM-TB representa, portanto, uma limitação estrutural do campo que esta revisão evidencia.

Essa sobreposição é reconhecida na literatura de sistemas de saúde. Penchansky e Thomas definem acesso como um conceito multidimensional que abrange disponibilidade, acessibilidade, capacidade financeira, acomodação e aceitabilidade^(33)^, dimensões que aparecem de forma fragmentada nas diferentes operacionalizações de cobertura identificadas nesta revisão, reforçando a necessidade de maior precisão conceitual no campo.

Desde dezembro de 2010, a OMS recomenda a utilização do TRM-TB como método diagnóstico primário da TB^(2,31)^, no entanto, observou-se que, em dois estudos, a utilização do teste foi vinculada a um resultado negativo da baciloscopia. Isso pode ter afetado o resultado da cobertura nesses locais, visto que na Guatemala, onde foi utilizado este critério, o TRM-TB sozinho resultou em um aumento de 12,3% nas notificações de TB pulmonar^(21)^. Apesar da sua larga adoção para diagnóstico da TB, a baciloscopia possui uma sensibilidade relativamente baixa em comparação ao TRM-TB, o qual é capaz de fornecer resultados muito mais rapidamente e, consequentemente, a instituição terapêutica precoce^(3,4,14,32)^.

Um estudo realizado no estado de São Paulo, Brasil, também apontou que a cobertura de realização da baciloscopia superou o TRM-TB entre as pessoas vivendo com HIV, alcançando uma cobertura de 70%^(28)^, ou seja, mesmo com a alta cobertura de TRM-TB, a baciloscopia tem sido utilizada de forma complementar ao teste, o que aumenta o número de subnotificação, sobretudo entre paucibacilares, como pessoas vivendo com HIV^(5)^, como também pode resultar na realização de tratamentos sem confirmação bacteriológica.

Sabe-se que o risco de adoecimento por TB é 23 vezes maior em pessoas vivendo com HIV^(34)^, dessa forma, reforça- se a necessidade da continuidade da utilização do TRM-TB alinhada a estratégias robustas que fortaleçam a busca ativa e o acompanhamento dessas pessoas.

Com base nesses achados, desafios supracitados no acesso ao diagnóstico pelo TRM-TB refletem barreiras estruturais e operacionais em serviços assistenciais, sobretudo de usuários coinfectados com TB e HIV, o que reforça a necessidade de estratégias contextualizadas e intervenções de melhoria da qualidade para otimizar a implementação do TRM-TB nessa população específica.

O TRM-TB também possui a capacidade de detectar bacilos mortos e/ou inativos, dessa forma é contraindicado para pessoas que estão em retratamento de TB^(2)^, no entanto, no estudo realizado na Rússia, em Belgorod, além do diagnóstico de novos adoecimentos, o TRM-TB também foi utilizado para a detecção de recidivas da doença^(14)^, sinalizando um possível uso inadequado de recursos, com potenciais reflexos na gestão de serviços, programas e políticas públicas. Situações semelhantes foram observadas no estudo realizado na Coreia do Sul, onde foi apontado o uso do teste em pacientes previamente tratados^(31)^.

Independentemente do critério adotado, a cobertura do TRM-TB variou nos diferentes locais de estudo, com alguns deles apontando mudanças positivas no decorrer dos anos. Os resultados corroboram o que vem sendo divulgado pela OMS, onde em 2024, houve uma variação substancial na cobertura de testes rápidos entre regiões e países. Entre as regiões, os melhores níveis de cobertura foram alcançados na Região Europeia (77%) e na Região do Pacífico Ocidental (70%), enquanto a menor cobertura foi identificada na Região do Sudeste Asiático (41%). Em relação aos países, 69 alcançaram níveis de cobertura de pelo menos 80% em 2024, mas em 21 países a cobertura foi inferior a 20%^(2)^. Vale ressaltar que a baixa cobertura pode ser um indicador de TB subdiagnosticada, sobretudo a TB-DR^(16,22)^.

A Estratégia End TB da OMS estabelece como meta a eliminação da tuberculose como problema de saúde pública até 2035, com redução de 95% das mortes e 90% da incidência em relação a 2015^(35)^. Os dados evidenciam que o alcance dessas metas depende não apenas da disponibilização do TRM-TB, mas da superação das barreiras estruturais, organizacionais e conceituais identificadas nesta revisão.

A baixa cobertura do TRM-TB esteve associada principalmente a dificuldades socioeconômicas e estruturais dos diferentes sistemas de saúde, além disso, observa-se a falta de diretrizes nacionais ou regionais para a implementação de testes moleculares em programas de TB ao redor do mundo^(20)^. Em 2015, um estudo que acompanhou a implementação do TRM-TB em 22 países sinalizou que apenas o Brasil e a Rússia, naquele momento, financiaram todos os testes com recursos domésticos, enquanto a maioria dos outros países dependia em algum grau de alguns dos 16 grupos de doadores internacionais identificados^(36)^.

Embora os suportes financeiro e de recursos materiais advindos de países terceiros para o incremento do diagnóstico de TB devam ser encorajados, vale ressaltar que maximizar os efeitos e a sustentabilidade a longo prazo do TRM-TB depende também da liderança governamental de saúde local, planejamento estratégico, coordenação de parceiros técnicos e doadores, monitoramento e avaliação contínuos. Para a implementação em larga escala é necessária a adoção de revisão de algoritmos, políticas, registros, formulários de solicitação e métodos de monitoramento e avaliação nacionais^(30)^.

Observou-se percepções similares com relação ao acesso aos serviços de diagnóstico para TB, incluindo o TRM-TB, além de disparidades importantes em regiões de um mesmo país. Um estudo realizado no Nepal sobre barreiras no acesso identificou que as principais dificuldades enfrentadas pela população são relacionadas a barreiras geográficas como a longa distância dos serviços, as más condições das estradas, a disponibilidade de meios de transporte e os custos associados à viagem, além disso, a falta de pessoal de saúde treinado em unidades de saúde localizadas em áreas periféricas é frequentemente relatada^(37)^.

As questões supracitadas apontam que, além de serem geograficamente inacessíveis, as instalações diagnósticas estão constantemente sobrecarregadas^(25)^. Portanto, barreiras geográficas provocam um efeito cascata nos indicadores de saúde para a TB, por exemplo, as longas distâncias até as instalações dificultam o diagnóstico, a taxa de notificação, o início do tratamento e, consequentemente, a sua continuidade^(38)^.

Uma revisão sistemática apontou que o baixo conhecimento sobre a TB entre a população é o principal aspecto que interfere no diagnóstico e tratamento, pois a maioria das pessoas não possui conhecimento suficiente sobre o que fazer quando apresentam alguns sintomas e como obter cuidados e tratamento adequados, especialmente nas áreas rurais^(39)^. Sob a perspectiva dos autores, vale destacar o fator socioeconômico como uma importante característica de disparidade entre as populações; isso reflete em como a TB ainda é uma doença com elevado estigma social.

Variações regionais também foram encontradas em um estudo realizado em Gana, onde foram observadas disponibilidade inadequada de equipamentos/suprimentos de diagnóstico no país, com proporções de locais de diagnóstico significativamente diferentes com valores variando de 32.531 pessoas por unidade de saúde em uma região, para 163.317 por unidade em outras^(38)^.

Faz-se necessária a presença de serviços diagnósticos descentralizados, já que sua falta impacta diretamente no acúmulo de pessoas acometidas por TB nos principais centros, impedindo, portanto, o acesso a resultados rápidos de diagnóstico^(39)^. Portanto, é preciso que os recursos advindos dos programas nacionais de TB não se concentrem em apenas adquirir máquinas de TRM-TB, mas para além disso, é preciso planejamento de gestão para que essas ferramentas estejam disponíveis à população de forma acessível, visando a equidade na oferta de serviços de saúde.

Além disso, é importante destacar que a incorporação do TRM-TB pode ser limitada por fatores organizacionais e pela manutenção de práticas diagnósticas previamente estabelecidas, mesmo em contextos com alta capacidade tecnológica^(31)^. Esse resultado sugere que a cobertura do TRM-TB não depende apenas da disponibilidade do teste, mas também da estrutura dos serviços, dos fluxos assistenciais e da capacitação das equipes. Assim, persistem desafios para a ampliação do uso do TRM, especialmente em cenários com elevada demanda e heterogeneidade regional.

O presente estudo possui limitações relacionadas à metodologia da revisão de escopo, como a não realização de avaliação crítica das evidências relatadas nos estudos incluídos, o que pode limitar a interpretação dos resultados, além da heterogeneidade de diferentes metodologias empregadas. Além disso, os resultados devem ser generalizados com cautela, uma vez que abordaram contextos específicos que possuem realidades e sistemas de saúde distintos e com diferentes incidências para TB.

A variação de critérios de cobertura entre os estudos indica baixa padronização conceitual no campo e evidencia a necessidade de maior clareza metodológica e terminológica, especialmente aqueles que mensuram aspectos do acesso e cobertura do TRM-TB mesmo que de forma secundária, de modo a evitar a subidentificação de evidências relevantes.

Os resultados desta revisão, portanto, apresentam como a cobertura tem sido medida e reportada em diferentes estudos e contextos, e quais dimensões acerca da utilização do teste têm sido priorizadas nestes cenários. Apesar destas diferenças metodológicas limitarem a comparabilidade direta entre países, fornecem um panorama importante sobre as estratégias locais de implementação do TRM-TB e possíveis nós críticos nas redes de atenção à TB.

Portanto, esta revisão fornece um panorama da cobertura do TRM-TB e identifica barreiras geográficas, estruturais e socioeconômicas que afetam o diagnóstico; evidencia a necessidade de padronização conceitual da cobertura e reforça a importância do TRM-TB para diagnóstico precoce, especialmente em populações em situações de vulnerabilidade. Os achados subsidiam a implementação de estratégias contextualizadas, capacitação de equipes, integração com práticas tradicionais e promoção da equidade, apoiando políticas baseadas em evidências, melhoria do cuidado e redução da subnotificação da TB.

## CONCLUSÃO

Os achados desta revisão revelaram que, de acordo com a literatura analisada, a cobertura do TRM-TB tem sido operacionalizada e descrita de maneiras distintas em diferentes países e contextos, abrangendo desde o percentual de pessoas com TB testadas inicialmente com métodos moleculares até indicadores de uso em subgrupos específicos, disponibilidade de equipamentos e acessibilidade geográfica.

Os estudos indicam avanços importantes na incorporação do TRM-TB como método diagnóstico, especialmente em países de alta carga de TB, embora em muitos cenários a utilização permaneça aquém das metas internacionais estabelecidas pela OMS. Os resultados sugerem que a baixa cobertura está fortemente associada a barreiras estruturais e socioeconômicas, dependência de financiamento externo, ausência de diretrizes nacionais claras para implementação de testes moleculares e insuficiente descentralização dos serviços de diagnóstico.

Para que o TRM-TB alcance seu potencial completo de diagnóstico e impacte positivamente no controle da TB, é necessário um compromisso renovado dos governos nacionais para a descentralização dos serviços, disponibilidade e capacitação de profissionais, bem como o desenvolvimento de políticas locais e regionais. O reforço no financiamento, a eliminação das barreiras de acesso e uma abordagem mais ambiciosa para ampliação sustentada da cobertura são passos essenciais para reduzir as iniquidades e avançar no diagnóstico efetivo da TB. Especial atenção deve ser dada às populações em situação de vulnerabilidade, priorizando estratégias que reduzam o estigma, ampliem o diagnóstico precoce e diminuam a subnotificação. No campo da pesquisa, recomenda-se a adoção de critérios padronizados e alinhados às diretrizes da OMS para mensurar a cobertura do TRM-TB, possibilitando comparações entre países e o monitoramento contínuo das metas estabelecidas.

## Data Availability

Todo o conjunto de dados que dá suporte aos resultados deste estudo foi publicado no próprio artigo.

# Translation: Implementación de la cobertura de la prueba rápida molecular para la tuberculosis en diferentes contextos internacionales: revisión de alcance

**DOI:** 10.1590/1980-220X-REEUSP-2026-0172es

**Authors:** Diogo Henrique Mendes da Silva, Ana Beatriz Marques Valença, Melisane Regina Lima Ferreira, Mariana Gaspar Botelho Funari de Faria, Rubia Laine de Paula Andrade, Rebeca Sousa Braga, Aline Aparecida Monroe

**Affiliations:** 1 Universidade de São Paulo, Escola de Enfermagem de Ribeirão Preto, Programa de Pós-Graduação Enfermagem em Saúde Pública, Ribeirão Preto, SP, Brasil.; 2 Ministério da Saúde, Secretaria de Vigilância em Saúde e Ambiente, Coordenação-Geral de Vigilância da Tuberculose, Micoses Endêmicas e Micobactérias Não Tuberculosas, Brasília, DF, Brasil.; 3 Universidade de São Paulo, Escola de Enfermagem de Ribeirão Preto, Departamento de Enfermagem Materno-Infantil e Saúde Pública, Ribeirão Preto, SP, Brasil.

**Keywords:** Tuberculosis, Técnicas de Diagnóstico Molecular, Pruebas de Diagnóstico Rápido.

## Abstract

**Objetivo:**

Realizar un mapeo de los estudios sobre la cobertura de las pruebas moleculares rápidas para la tuberculosis en diferentes contextos internacionales.

**Método:**

Revisión de alcance, realizada según las recomendaciones de PRISMA-ScR, con búsquedas en las bases de datos MEDLINE, Scopus, Web of Science, Embase, LILACS y BDTD, que abarcan el período de 2011 a 2025.

**Resultados:**

Los 20 estudios incluidos mostraron heterogeneidad conceptual en la definición operativa de la cobertura, que abarcó desde proporciones de pruebas en grupos poblacionales específicos hasta indicadores de disponibilidad de equipos y accesibilidad geográfica, sin estandarización entre países y contextos. Los valores variaron de menos del 1 % a aproximadamente el 80 %, con diferencias significativas entre países, poblaciones objetivo y períodos analizados. Los desafíos socioeconómicos, estructurales, financieros y de acceso a las instalaciones dificultan el uso de la prueba.

**Conclusión:**

La cobertura de las pruebas varía entre países y contextos, limitada por barreras estructurales y financieras, así como por la falta de estandarización conceptual y metodológica en su medición. Ampliar y monitorear la cobertura de manera comparable exige compromiso gubernamental, descentralización y la adopción de criterios uniformes alineados con las directrices de la Organización Mundial de la Salud.

## INTRODUCCIÓN

Aunque es curable, la tuberculosis (TB) sigue representando un desafío para los sistemas de salud en todo el mundo. El elevado número de personas que enferman en los países en desarrollo refuerza la necesidad de continuar con las estrategias de prevención y control^(1)^. Se estima que, para el año 2025, más de 10 millones de personas seguirán enfermando de TB cada año y más de un millón morirán a causa de la enfermedad^(2)^.

El examen de baciloscopia, aunque es una técnica de diagnóstico sencilla y de bajo costo, presenta limitaciones, como baja sensibilidad y especificidad^(3,4)^. En un principio, estas limitaciones se superaban mediante cultivos microbiológicos, considerados el estándar de referencia para el diagnóstico de la tuberculosis, la tuberculosis resistente a los medicamentos (TB-DR) y las micobacterias no tuberculosas^(3)^. Sin embargo, la principal desventaja del cultivo es el tiempo prolongado que se tarda en obtener los resultados, normalmente entre cuatro y ocho semanas^(5)^.

Debido a estas limitaciones, se desarrollaron nuevos métodos de diagnóstico para mejorar la detección de la enfermedad y avanzar hacia el cumplimiento de los Objetivos de Desarrollo Sostenible (ODS), adoptados en septiembre de 2015, relacionados con la respuesta social y de salud para combatir la tuberculosis^(6)^.

En este sentido, entre las pruebas moleculares desarrolladas, la tecnología GeneXpert MTB/RIF, conocida en Brasil como Prueba Molecular Rápida para la Tuberculosis (TRM-TB), fue recomendada a nivel mundial por la Organización Mundial de la Salud (OMS) a partir de diciembre de 2010^(5)^, la cual posteriormente se perfeccionó y fue sustituida por el Xpert MTB/RIF Ultra en 2019, una versión con mayor sensibilidad y especificidad^(7)^. Ambas pruebas tienen la capacidad de detectar el complejo *Mycobacterium tuberculosis* e identificar cepas resistentes a la rifampicina en muestras biológicas en aproximadamente dos horas^(3,4,7)^.

Tras la recomendación, varios países comenzaron a adoptar la tecnología para el diagnóstico de nuevos casos de tuberculosis. Sin embargo, estudios realizados en condiciones de atención de rutina muestran que el TRM-TB aumentó la detección de casos y mejoró el acceso al diagnóstico^(5,8)^.

A pesar de las recomendaciones para implementar la prueba en diferentes contextos, persisten brechas no solo en cuanto a su aplicación práctica en los sistemas de salud, sino sobre todo en cuanto a la forma en que se define y mide su cobertura. La ausencia de criterios estandarizados para operacionalizar la cobertura del TRM-TB dificulta la comparación entre países y la evaluación del alcance de las metas establecidas por la OMS, lo que constituye una brecha científica que esta revisión se propone mapear.

Para comprender este vacío de conocimiento, es importante señalar que la cobertura, el acceso, la utilización y la disponibilidad son conceptos distintos, aunque con frecuencia se utilizan de manera superpuesta en la literatura. La cobertura, en sentido amplio, expresa el grado de interacción entre el servicio y la población a la que está destinado, desde la asignación de recursos hasta la obtención del resultado deseado^(9)^.

Ante esto, este estudio tiene como objetivo mapear estudios sobre la cobertura del TRM-TB en diferentes contextos internacionales, identificando cómo se ha operacionalizado y descrito este indicador en la literatura, con el fin de respaldar estrategias de gestión y políticas de salud orientadas al control de la tuberculosis.

## MÉTODO

### Tipo de Estudio

Se trata de una revisión de alcance, realizada a partir de las recomendaciones de la *lista de verificación «Preferred Reporting Items for Systematic Reviews and Meta-Analyses: extension for Scoping Reviews»* (PRISMA-ScR)^(10)^, para orientar la presentación de los resultados. La realización siguió el manual del JBI^(11)^, que establece las etapas metodológicas de la revisión de alcance: 1) definición de la pregunta de investigación; 2) búsqueda de los estudios relevantes; 3) selección y evaluación preliminar de los estudios; 4) análisis de la información de los estudios; y 5) organización, síntesis y presentación de los resultados. Se utilizó el PRISMA-ScR como guía de transparencia en la presentación de cada una de estas etapas. El protocolo de esta revisión se registró en la base de datos *Open Science Framework* (OSF) con el DOI: https://doi.org/10.17605/OSF.IO/6EUH7.

### Recolección de Datos

La pregunta guía «¿Cómo se ha implementado y descrito la cobertura del TRM-TB para el diagnóstico de personas con tuberculosis o con signos y síntomas sugestivos de tuberculosis en diferentes contextos internacionales?» se desarrolló con base en la metodología mnemotécnica Población, Concepto y Contexto (PCC). En este modelo, la Población (P) estuvo representada por las personas con tuberculosis o con signos y síntomas indicativos de tuberculosis; el Concepto (C) correspondió a la cobertura del TRM-TB; y el Contexto (C) abarcó los diferentes contextos internacionales.

A partir de esta pregunta, este estudio consideró la concepción ampliada de la cobertura de los servicios de salud propuesta por Tanahashi, entendida como el grado de interacción entre el servicio y la población a la que está dirigido, abarcando desde la disponibilidad y la asignación de recursos hasta la obtención del resultado deseado^(9)^. Teniendo en cuenta las dimensiones de disponibilidad, accesibilidad, aceptabilidad, contacto y efectividad, se consideraron como indicadores de cobertura la proporción de pruebas realizadas en grupos poblacionales, la disponibilidad de equipos en los servicios de salud y la accesibilidad geográfica a las instalaciones de diagnóstico, de acuerdo con la operacionalización presentada en cada estudio. Dichas dimensiones, consideradas como indicadores de cobertura, se identificaron a partir de la lectura de los estudios incluidos.

Para la recopilación de los estudios relevantes, se realizó una búsqueda electrónica en las siguientes bases de datos: *Scopus, Web of Science, Medical Literature Analysis and Retrieval System Online* (MEDLINE), Literatura Latinoamericana y del Caribe en Ciencias de la Salud (LILACS) y *Embase*. También se realizaron búsquedas para identificar la literatura gris en la Biblioteca Digital Brasileña de Tesis y Disertaciones (BDTD).

La búsqueda electrónica se llevó a cabo en diciembre de 2025 utilizando palabras clave identificadas previamente en la literatura y descriptores indexados en la plataforma de Descriptores en Ciencias de la Salud (DeCS), así como sus sinónimos en portugués y español. Asimismo, se utilizaron descriptores indexados en la plataforma *The Medical Subject Headings* (MeSH) para la elaboración de las estrategias de búsqueda en inglés. Para relacionar los descriptores y las palabras clave entre sí, se aplicaron los operadores booleanos *AND* y *OR.* Los descriptores, las palabras clave, el período y las estrategias de búsqueda realizadas para cada base de datos se presentan en el Cuadro 1. La estrategia de búsqueda fue validada por los coautores, miembros del mismo grupo de investigación. Los filtros de idioma (inglés, portugués y español) se aplicaron en la etapa de selección de los estudios.

**Cuadro 1:** Estrategias utilizadas para la búsqueda de estudios aplicadas a cada base de datos – Ribeirão Preto, SP, Brasil, 2025.

Bases de datos	Estrategias de búsqueda
MEDLINE	*((“tuberculosis” [MeSH Terms] OR “tuberculosis” [All Fields] OR “tuberculoses” [All Fields] OR “tb” [All Fields]) AND (“xpert” [All Fields] OR “genexpert” [All Fields] OR “gene xpert” [All Fields] OR “cepheid” [All Fields] OR “cepheids” [All Fields] OR “rapid molecular test” [All Fields]) AND (“coverage” [All Fields] OR “coverages” [All Fields] OR “diagnostic coverage” [All Fields])) AND (medline[Filter]) AND (2011:2025[pdat])*
Scopus	*(TITLE-ABS-KEY (tuberculosis OR tb) AND TITLE-ABS-KEY (xpert OR genexpert OR “gene xpert” OR cepheid OR “rapid molecular test”) AND TITLE-ABS-KEY (coverage OR "diagnostic coverage") ) AND PUBYEAR > 2010 AND PUBYEAR < 2026*
Web of Science	*tuberculosis OR tb (Topic) and xpert OR genexpert OR “gene xpert” OR cepheid OR “rapid molecular test” (Topic) and coverage OR “diagnostic coverage” (Topic) and 2025 or 2024 or 2023 or 2022 or 2021 or 2020 or 2019 or 2018 or 2017 or 2016 or 2015 or 2014 or 2013 or 2012 or 2011 (Publication Years)*
Embase	*#1 (’tuberculosis‘/exp OR tuberculosis OR ’tb‘/exp OR tb) AND [2011-2025]/py #2 xpert OR genexpert OR ’gene xpert‘ OR cepheid OR ’rapid molecular test‘ #3 coverage OR ’diagnostic coverage‘ #4 #1 AND #2 AND #3*
LILACS	*(tuberculosis OR tuberculose OR tb) AND (xpert OR genexpert OR “gene xpert” OR cepheid OR “rapid molecular test” OR “teste rápido molecular” OR trm-tb OR "teste molecular rápido” OR “prueba rápida molecular” OR prm OR “prueba molecular rápida”) AND (coverage OR "diagnostic coverage" OR cobertura OR “cobertura diagnóstica” OR "cobertura de diagnóstico”) AND (year_cluster:[2011 TO 2025])*
Biblioteca Digital Brasileira de Teses e Dissertações	*“(Todos os campos:tuberculosis OR tuberculose OR tb E Todos os campos:xpert OR genexpert OR “gene xpert” OR cepheid OR “rapid molecular test” OR “teste rápido molecular” OR trm-tb OR “teste molecular rápido” OR “prueba rápida molecular” OR prm OR “prueba molecular rápida” E Todos os campos:coverage OR “diagnostic coverage” OR cobertura OR “cobertura diagnóstica” OR “cobertura de diagnóstico”)”*

LILACS: Literatura latinoamericana y del Caribe en ciencias de la salud.

### Criterios de Selección

Los criterios de elegibilidad para esta revisión de alcance incluyeron estudios que abordaron la cobertura o el porcentaje de uso del TRM-TB (GeneXpert MTB/RIF y/o Xpert MTB/RIF Ultra), entendidos como indicadores cuantitativos de uso (cobertura basada en el uso del TRM-TB entre personas con signos y síntomas sugestivos de tuberculosis; cobertura basada en el uso del TRM-TB entre las personas diagnosticadas con tuberculosis; cobertura basada en el uso del TRM-TB en personas con tuberculosis resistente a los fármacos (TB-DR); cobertura basada en el uso del TRM-TB en personas que viven con VIH; y cobertura basada en el uso del TRM-TB entre las personas que dieron negativo en la baciloscopia de esputo) y/o disponibilidad o acceso a la prueba (cobertura basada en el número de máquinas de TRM-TB en relación con el número de centros de salud; y cobertura del TRM-TB basada en la distancia de la población a las instalaciones de diagnóstico). Así, se incluyeron estudios que presentaran estos indicadores como objetivo principal o como hallazgo secundario, siempre que los datos cuantitativos estuvieran claramente descritos; que tuvieran en la población analizada a personas con tuberculosis o con signos y síntomas sugestivos de tuberculosis, independientemente de la forma clínica o las condiciones de vulnerabilidad; y que se hubieran publicado entre 2011 y 2025.

La decisión de considerar estos estudios se debe a su amplia difusión en programas nacionales e internacionales de control de la TB, a su reconocimiento por parte de la OMS como referencia para el diagnóstico molecular rápido y a la mayor disponibilidad de estudios que adoptan métricas comparables. Esta delimitación buscó reducir la heterogeneidad entre distintas tecnologías y permitir una mayor consistencia en el análisis de la cobertura. La elección del período se basa en la recomendación adoptada por la OMS para el uso de la prueba TRM-TB en la detección de la tuberculosis y la tuberculosis resistente a múltiples fármacos (TB-DR), a partir de diciembre de 2010^(2)^.

Se consideraron elegibles los estudios publicados en inglés, portugués y español, primarios o secundarios, revisados por pares o no, siempre que en su alcance se incluyera algún indicador de cobertura del TRM-TB en cualquier zona geográfica del mundo, utilizando cualquier enfoque y diseño metodológico. Se consideraron tesis, disertaciones, trabajos de fin de carrera y resúmenes de congresos.

Se excluyeron protocolos, manuales, directrices, notas técnicas, cartas, editoriales y comentarios, por no presentar datos empíricos cuantitativos sobre el uso o la cobertura de la prueba objeto de esta revisión. Además, los estudios que no presentaran en su título y resumen información clara relacionada con la cobertura o el TRM-TB fueron descartados ya en la etapa de selección de títulos y resúmenes. También se excluyeron los estudios que incluían otros tipos de pruebas de diagnóstico molecular distintas de GeneXpert MTB/RIF o Xpert MTB/RIF Ultra, ya que involucraban tipos de muestras y procedimientos de laboratorio no comparables con el TRM-TB definido para esta revisión.

### Extracción y Análisis de Datos

Tras el proceso de búsqueda en las bases de datos, las publicaciones se exportaron a la plataforma *Rayyan AI*, una herramienta de apoyo a las revisiones sistemáticas que permite identificar y eliminar duplicados, así como seleccionar los estudios elegibles^(12)^.

La extracción se llevó a cabo en dos partes: la primera fue realizada de manera independiente por dos revisores, quienes evaluaron los títulos y resúmenes de las referencias identificadas, seleccionando los estudios potencialmente elegibles con base en la pregunta de investigación y los criterios de elegibilidad. Las discrepancias fueron resueltas por un tercer autor calificado. La segunda parte consistió en la lectura y evaluación completa de los estudios preseleccionados, documentando los motivos de exclusión en un diagrama de flujo basado en PRISMA-ScR^(10)^.

La información de los estudios incluidos se extrajo a partir de un instrumento adaptado para su uso en una hoja de cálculo de Excel que contenía las siguientes variables: autor(es); título; revista; tipo de publicación; idioma del estudio; país donde se realizó el estudio; año de publicación; objetivo; diseño del estudio; período del estudio; población del estudio; tamaño de la muestra; definición operativa de la cobertura del TRM-TB; principales resultados relacionados con la cobertura y/o porcentaje de uso de la prueba; población a la que está destinada la prueba; y principales discusiones y conclusiones de los autores.

De estas variables, se presentaron aquellas directamente relacionadas con la pregunta de investigación: definición operativa de la cobertura, valores de cobertura medidos, diseño del estudio y país donde se llevó a cabo. Las demás variables se utilizaron para orientar la síntesis narrativa y la construcción de las categorías analíticas.

Todos los resultados se presentaron en forma de una tabla descriptiva que contenía la definición operativa de cobertura utilizada y los valores de cobertura medidos en los estudios. Además, se enumeraron dos categorías de análisis para componer la discusión de los resultados: factores asociados a la baja cobertura y acceso al diagnóstico mediante el TRM-TB. Estas categorías surgieron durante la lectura y el análisis de los estudios incluidos, a partir de los temas recurrentes identificados en sus discusiones y conclusiones.

### Aspectos Éticos

Esta revisión de alcance utilizó exclusivamente fuentes de literatura secundaria de acceso público, sin recolección de datos primarios ni información personal identificable. Por esta razón, y de conformidad con las normas éticas aplicables, no fue necesaria la aprobación de un Comité de Ética en Investigación ni la obtención de un Consentimiento Libre e Informado.

## RESULTADOS

Los estudios incluidos se analizaron centrándose en la forma en que se puso en práctica y se describió la cobertura del TRM-TB en cada contexto. Se identificaron 502 registros en las bases de datos. Tras eliminar los estudios duplicados, se procedió a la lectura de los títulos y resúmenes de 220 de ellos. Una vez realizada la lectura, la presente revisión incluyó 20 estudios que cumplían con los criterios de inclusión y respondían a la pregunta de investigación. Los motivos de exclusión de los artículos leídos en su totalidad se documentaron tal como se muestra en el diagrama de flujo de la Figura 1.

**Figura 1:** Diagrama de flujo de inclusión y exclusión de las publicaciones de acuerdo con las recomendaciones de PRISMA-ScR – Ribeirão Preto, Brasil, 2025. LILACS: Literatura Latinoamericana y del Caribe en Ciencias de la Salud; TRM-TB: Prueba Rápida Molecular para la Tuberculosis.

El período de publicación de los estudios incluidos fue de 2017 a 2025^(13,14,15,16,17,18,19,20,21,22,23,24,25,26,27,28,29,30,31,32)^, con mayor concentración en 2020^(19,20,21)^, 2021^(22,23,24)^ y 2024^(28,29,30)^, años en los que se identificaron tres publicaciones cada uno. En cuanto al idioma de publicación, 19 estudios estaban disponibles en inglés^(13,14,15,16,17,18,19,21,22,23,24,25,26,27,28,29,30,31,32)^ y uno en español^(20)^. En cuanto al diseño de los estudios, se observó un predominio de enfoques cuantitativos, con solo un estudio que utilizó métodos mixtos^(22)^. En su mayoría, se trataba de estudios de cohorte^(16,17,19,24)^ (Cuadro 2). Entre los estudios incluidos, se identificaron siete formas distintas de operacionalizar la cobertura del TRM-TB, categorizadas a partir de la información disponible, a saber: 1) cobertura basada en el uso del TRM-TB entre las personas con signos y síntomas sugestivos de tuberculosis (n = 8)^(15,17,18,24,26,27,29,30)^; 2) cobertura basada en el uso del TRM-TB entre las personas diagnosticadas con tuberculosis (n = 4)^(13,14,19,20)^; 3) cobertura basada en el uso del TRM-TB en personas con tuberculosis resistente a múltiples fármacos (n = 2)^(16,31)^; 4) cobertura basada en el uso del TRM-TB en personas que viven con VIH (n = 2)^(28,32)^; 5) cobertura basada en el uso del TRM-TB entre las personas con resultado negativo en el examen de baciloscopia de esputo (n = 2)^(21,23)^; 6) cobertura basada en el número de equipos de TRM-TB en relación con el número de unidades de salud (n = 1)^(22)^; y 7) cobertura del TRM-TB basada en la distancia de la población a los centros de diagnóstico (n = 1)^(25)^ (Cuadro 2).

**Cuadro 2:** Presentación de los estudios incluidos en la revisión – Ribeirão Preto, SP, Brasil, 2025.

Autor, año y país	Tipo de publicación	Diseño del estudio	Objetivos del estudio	Definición operativa de la cobertura	Valores de cobertura de la prueba molecular rápida
Rees et al., 2017^(13)^; Sudáfrica	Artículo	Estudio retrospectivo	Evaluar las tendencias diagnósticas en pacientes tratados por tuberculosis en un subdistrito rural de Sudáfrica, en relación con la implementación del TRM-TB y su algoritmo correspondiente, utilizando datos de rutina.	Cobertura basada en la uso del TRM-TB entre las personas diagnosticadas con TB.	Del 0% en 2010 al 69,9% en 2015 en el subdistrito de Greater Tzaneen, Sudáfrica.
Zemlyansky et al., 2017^(14)^; Rusia	Artículo	Estudio transversal	Evaluar la eficacia y desarrollar un modelo óptimo para el diagnóstico de laboratorio de la tuberculosis utilizando el TRM-TB.	Cobertura basada en la uso del TRM-TB entre las personas diagnosticadas con TB.	El 75,3% entre los nuevos casos y el 80,8% en las recidivas en la región de Belgorod, Rusia, en 2015.
Awan et al., 2018^(15)^; Pakistán	Artículo	Estudio transversal	Cubrir las lagunas existentes en la literatura sobre las posibles limitaciones en la implementación del TRM-TB en entornos programáticos con una alta carga de TB-DR.	Cobertura basada en el uso del TRM-TB entre las personas con signos y síntomas que sugieren de tuberculosis.	44,3% entre julio de 2013 y junio de 2015 en Karachi, Pakistán.
Evans et al., 2018^(16)^; Sudáfrica	Artículo	Estudio de cohorte retrospectivo	Analizar el impacto de la introducción del TRM-TB y de la descentralización del tratamiento en la retención en el tratamiento y en los resultados del tratamiento de la TB-DR en Johannesburgo, Sudáfrica.	Cobertura basada en el uso del TRM-TB en personas con TB-DR.	43,4% entre julio de 2011 y junio de 2012 y 60,5% entre julio de 2013 y junio de 2014, en Johannesburgo, Sudáfrica.
Farr et al., 2019^(17)^; Uganda	Artículo	Estudio de cohorte prospectivo	Caracterizar en qué medida los pacientes con tuberculosis en Uganda recibían atención de acuerdo con las directrices nacionales e internacionales.	Cobertura basada en el uso del TRM-TB entre las personas con signos y síntomas sugestivos de tuberculosis.	19,5% en 2017 en 24 centros de salud de Uganda.
Kabasakalyan et al., 2019^(18)^; Armenia	Artículo	Estudio observacional retrospectivo	Evaluar el cambio en el perfil diagnóstico de laboratorio de *Mycobacterium tuberculosis* tras la introducción del TRM-TB.	Cobertura basada en el uso del TRM-TB entre las personas con signos y síntomas de e que sugieran de tuberculosis.	58% en el periodo comprendido entre 2013 y 2017 en Armenia.
Wu et al., 2020^(19)^; China	Artículo	Estudio de cohorte retrospectivo	Evaluar el efecto de la implantación del TRM-TB en la detección de casos de tuberculosis pulmonar.	Cobertura basada en el uso del TRM-TB entre las personas diagnosticadas con TB.	del 54% en el primer semestre de 2018 en Shanghái, China.
Agredo and Osorio, 2020^(20)^; Colombia	Artículo	Estudio descriptivo	Describir la cobertura y la adherencia al tratamiento con TRM-TB en pacientes con tuberculosis pulmonar en una ciudad con una elevada carga de la enfermedad en Colombia.	Cobertura basada en la uso del TRM-TB entre las personas diagnosticadas con TB.	10,35% entre 2013 y 2019 en el municipio de Cali, Colombia.
Creswell et al., 2020^(21)^; Guatemala	Artículo	Estudio de evaluación programática	Evaluar el impacto de la introducción de la prueba TRM-TB como prueba de seguimiento tras el frotis microscópico en el número total de notificaciones de tuberculosis pulmonar.	Cobertura basada en el uso de la prueba TRM-TB entre las personas con resultado negativo en la baciloscopia de esputo.	39,6% entre abril de 2015 y marzo de 2016 en Guatemala.
Ntinginya et al., 2021^(22)^; Uganda, Kenia y Tanzania	Artículo	Cuantitativo y cualitativo	Investigar las barreras y oportunidades para maximizar la adopción y el uso de diagnósticos moleculares en entornos de atención sanitaria habitual.	Cobertura basada en el número de máquinas de TRM-TB en relación con el número de centros sanitarias.	De septiembre de 2016 a diciembre de 2017, el 74% en Uganda, el 39% en Tanzania y el 42% en Kenia.
Li et al., 2021^(23)^; China	Artículo	Estudio transversal de evaluación antes y después	Evaluar el impacto de la Fase III del Proyecto China-Gates contra la Tuberculosis en el acceso a diagnósticos y tratamientos de calidad para la tuberculosis.	Cobertura basada en el uso del TRM-TB entre las personas que dieron negativo en la a de baciloscopia del esputo.	0% en 2015, 16% en 2018 utilizando únicamente el TRM y 34,4% en 2018 utilizando el TRM-TB + cultivo, en 12 localidades de tres provincias de China: Zhejiang, Jilin y Ningxia.
Turaev et al., 2021^(24)^; Uzbekistán	Artículo	Estudio de cohorte prospectivo	Evaluar la cobertura del TRM-TB entre las personas con tuberculosis presunta, los factores asociados a ella y los retrasos que conlleva.	Cobertura basada en el uso del TRM-TB entre las personas con signos y síntomas que sugieren de tuberculosis.	24% en 2018 y 46% en 2019 en Uzbekistán.
Milaham et al., 2022^(25)^; Nigeria	Artículo	Estudio ecológico retrospectivo	Investigar la distribución espacial de las tasas de notificación de casos de tuberculosis, los diagnósticos y la cobertura entre las áreas de gobierno local.	Cobertura del TRM-TB en función de la distancia de la población a las centros de diagnóstico.	Entre 2017 y 2019, el 49% de la población del estado de Katsina (Nigeria) vivía a una distancia superior a 20 km de un centro de TRM-TB.
Ishengoma et al., 2023^(26)^; Tanzania	Resumen publicado en las actas del evento	Estudio descriptivo	Evaluar la eficacia de las clínicas móviles en Tanzania como estrategia para detectar casos de TB no diagnosticados en zonas de difícil acceso.	Cobertura basada en la uso del TRM-TB entre las personas con signos y síntomas e es que sugieren de tuberculosis.	el 34,5% en ocho regiones de Tanzania	mediante clínicas móviles.
Getahun et al., 2023^(27)^; Etiopía	Artículo	Estudio retrospectivo	Revisar los registros disponibles en centros de salud seleccionados de Adís Abeba, Etiopía, y evaluar el impacto del TRM-TB en el número de casos de tuberculosis detectados y en el éxito en los resultados del tratamiento.	Cobertura basada en el uso del TRM-TB entre las personas con signos y síntomas indicativos de tuberculosis.	71,1% entre 2015 y 2018 en Adís Abeba, Etiopía.
Faria et al., 2024^(28)^; Brasil	Artículo	Estudio ecológico	Analizar el diagnóstico de tuberculosis pulmonar no confirmada antes y después de la implantación del TRM-TB para personas que viven con el VIH en municipios del estado de São Paulo, Brasil.	Cobertura basada en el uso del TRM-TB en personas que viven con el VIH	Un 49,4% entre 2010 y 2020 en el estado de São Paulo, Brasil.
Pank et al., 2024^(29)^; Papúa-Nueva Guinea	Artículo	Estudio prospectivo de implementación	Evaluar la implementación inicial del «*Systematic Island-Wide Engagement & Elimination Project for TB*» en Daru mediante una iniciativa de mejora continua de la calidad.	Cobertura basada en el uso del TRM-TB entre las personas con signos y síntomas que sugieran de tuberculosis.	77,9% entre mayo y diciembre de 2023 en Daru, Papúa Nueva Guinea.
Dakulala et al., 2024^(30)^; Papúa-Nueva Guinea	Artículo	Estudio retrospectivo	Realización de un cribado de la población de la isla de Daru mediante una furgoneta móvil equipada con máquinas de TRM-TB y rayos X digitales con software de diagnóstico asistido por ordenador para la tuberculosis.	Cobertura basada en el uso del TRM-TB entre las personas con signos y síntomas de e que sugieran de tuberculosis.	79,7% entre febrero y octubre de 2018 y entre febrero y marzo de 2019, en Daru, Papúa Nueva Guinea, utilizando una furgoneta móvil.
Mok et al., 2025^(31)^; Corea del Sur	Artículo	Estudio de cohorte retrospectivo	Evaluar la cobertura de las pruebas moleculares de susceptibilidad a los fármacos entre pacientes con tuberculosis pulmonar multirresistente (TB-DR) en Corea del Sur e identificar los factores que influyen en su falta de aplicación.	Cobertura basada en el uso de la prueba de sensibilidad a los fármacos antituberculosos (TRM-TB) en personas con tuberculosis multirresistente (TB-DR).	22,1% del total de pacientes, de los cuales el 23,5% correspondía a casos nuevos y el 19,9% a pacientes previamente tratados en Corea del Sur.
Motlhaoleng et al., 2025^(32)^; Sudáfrica	Artículo	Estudio descriptivo	Evaluar la cascada asistencial del programa «*Targeted Universal Testing for TB*» para describir su implementación dentro de la atención continuada a las personas que viven con el VIH en centros sanitarios de alto volumen (≥6500 pacientes), volumen medio (entre 3500 y <6500 pacientes) y bajo (<3500 pacientes)	Cobertura basada en el uso del TRM-TB en personas que viven con el VIH	En el distrito rural de Zululand, las instalaciones de alto volumen registraron un 44,4% en 2022 y un 43,3% en 2023; las de volumen medio, un 43,1% y un 32,0%; y las de bajo volumen, un 55,0% y un 63,9%, respectivamente. En el municipio de eThekwini (distrito urbano), las instalaciones de alto volumen registraron un 22,3% en 2022 y un 28,3% en 2023; las de volumen medio, un 20,5%

TB: tuberculosis; TRM-TB: prueba rápida molecular para la tuberculosis; TB-DR: tuberculosis resistente a los medicamentos; VIH: *virus de la inmunodeficiencia humana*.

En cuanto a la operacionalización, las categorías difieren en cuanto al numerador, el denominador y la población objetivo considerados. En las categorías basadas en el uso de la prueba, el numerador correspondió al número de personas sometidas a la prueba TRM-TB, y el denominador varió según el grupo poblacional: personas con signos y síntomas sugerentes, personas diagnosticadas con TB, personas con TB-DR, personas que viven con VIH y personas con baciloscopia negativa.

En la categoría basada en la disponibilidad de equipos, el numerador correspondió al número de máquinas instaladas y el denominador, al número de centros de salud. En la categoría basada en la accesibilidad geográfica, el numerador correspondió a la población que residía a más de una distancia determinada de un centro de diagnóstico y el denominador, a la población total del área estudiada.

En cuanto al número de estudios por países donde se llevaron a cabo: Sudáfrica (n = 3)^(13,16,32)^, China (n = 2)^(19,23)^, Papúa Nueva Guinea (n = 2)^(29,30)^; Corea del Sur (n = 1)^(31)^, Armenia (n = 1)^(18)^, Uzbekistán (n = 1)^(24)^, Rusia (n = 1)^(14)^, Pakistán (n = 1)^(15)^, Etiopía (n = 1)^(27)^, Uganda (n = 1)^(17)^, Tanzania (n = 1)^(26)^, Nigeria (n = 1)^(25)^, Brasil (n = 1)^(28)^, Colombia (n = 1)^(20)^ y Guatemala (n = 1)^(21)^. También hubo un estudio que incluyó a tres países de África: Uganda, Kenia y Tanzania (n = 1)^(22)^.

A partir de la lectura de los estudios incluidos, surgieron dos categorías analíticas para organizar los principales hallazgos: factores asociados a la baja cobertura y al acceso al diagnóstico mediante la prueba TRM-TB.

### Factores Asociados a la Baja Cobertura

En los estudios que reportaron una baja cobertura del TRM-TB, se identificaron barreras para el uso de la prueba, incluyendo factores socioeconómicos y culturales, así como limitaciones estructurales de los sistemas de salud. Entre estas, se destacaron: baja búsqueda activa, calidad insuficiente de las muestras, uso del equipo restringido a un turno de trabajo, errores al completar las solicitudes, subfinanciamiento de la salud, dificultades de gestión entre administradores y profesionales, escasez de recursos humanos calificados y falta de infraestructura básica, como el suministro de agua y electricidad^(22,24,28,29,32)^.

Algunos estudios informaron el uso de máquinas de TRM-TB con solo cuatro módulos, lo que se asoció con un menor número de personas evaluadas y una menor detección, especialmente de la TB-DR; en esos contextos, se describieron experiencias con equipos de 16 módulos para ampliar la capacidad de pruebas^(25,26)^.

También se ha informado, en ciertos países, de la depen­dencia de presupuestos de salud financiados por donantes externos, con informes de que las fluctuaciones económicas en esos países afectaron la continuidad de la oferta de diagnóstico y tratamiento^(22)^. Además, un estudio mencionó las dificultades de los programas contra la tuberculosis para planificar intervenciones e inversiones basadas en estimaciones puntuales de la carga de tuberculosis de la OMS, sujetas a variaciones anuales y amplios intervalos de confianza^(21)^.

### Acceso al Diagnóstico Mediante TRM-TB

Algunos estudios describieron limitaciones en el acceso de la población a las unidades que ofrecen diagnóstico mediante TRM-TB. En el estado de Katsina, en Nigeria, la distribución de las instalaciones se consideró desigual, ya que el 100% de la población de dos Áreas de Gobierno Local (LGA) y el 75% de ocho LGA se encontraban a más de 20 km de cualquier centro de diagnóstico, lo que indica una mayor distancia que deben recorrer las personas que residen en esas áreas^(25)^. Además, se destacó la dificultad en materia de infraestructura y atención médica en las zonas rurales en comparación con las zonas urbanas^(32)^.

En Uganda, Tanzania y Kenia, el TRM-TB no se utilizaba de manera universal para todas las personas con signos y síntomas sugerentes de tuberculosis; su indicación era prioritaria para las personas que vivían con VIH o que habían tenido contacto previo con la tuberculosis resistente a múltiples fármacos (TRM-TB). A partir de 2019, la recomendación en esos países pasó a abarcar a todas las personas con signos y síntomas sugerentes de tuberculosis^(22)^.

En la cohorte realizada en Tashkent y Bujará, en Uzbekistán, se observaron diferencias regionales en el acceso a la prueba. La proporción de personas que no se sometieron a la prueba de detección de la tuberculosis (TRM-TB) fue mayor en Bujará (73%; IC 95%: 69–76) que en Tashkent (28%; IC 95%: 25–30) (p < 0,001).

En Bujará, el 51% de las personas con tuberculosis acudieron a centros sin servicio de diagnóstico, en comparación con el 9% en Tashkent; además, el 80% de los residentes de Bujará acudieron a centros rurales periféricos, mientras que el 88% de los residentes de Tashkent estaban vinculados a centros ubicados en zonas céntricas^(24)^.

El estudio realizado en Sudáfrica señala debilidades en el uso del TRM-TB entre las personas que viven con VIH en los distritos de Zululand (rural) y eThekwini (urbano), al considerar centros de alto volumen (≥6500 pacientes), volumen medio (de 3500 a <6500 pacientes) y bajo volumen (<3500 pacientes). La situación más crítica se da en el contexto urbano, especialmente en los centros de volumen medio y bajo^(32)^.

En doce localidades de tres provincias de China, entre 2015 y 2018, se observó un aumento en el acceso a diagnósticos de calidad para la tuberculosis, relacionado con el incremento del uso del TRM-TB y la expansión de los servicios de diagnóstico para personas con baciloscopia negativa^(23)^.

## DISCUSIÓN

A partir del análisis de las publicaciones, se observó un mayor número de estudios realizados en los continentes africano y asiático, lo que puede estar relacionado con el elevado número de casos y la alta incidencia de la tuberculosis en esos lugares. Geográficamente, la mayoría de las personas que desarrollaron tuberculosis en los últimos años se encontraban en las regiones del sudeste asiático (45%), África (24%) y el Pacífico occidental (17%), con proporciones menores en el Mediterráneo oriental (8,6%), las Américas (3,2%) y Europa (2,1%)^(2)^.

Un hallazgo central de esta revisión fue la heterogeneidad conceptual y operativa del término «cobertura» asociado a la prueba molecular rápida para la tuberculosis (TRM-TB). Aunque la OMS utiliza como criterio básico la proporción de personas con tuberculosis a las que se les realizó inicialmente una prueba molecular rápida^(2)^, solo cuatro de los estudios incluidos adoptaron esta definición de manera explícita^(13,14,19,20)^. Sin embargo, se observó que otras investigaciones evaluaron aspectos relacionados con la cobertura de la prueba, como la disponibilidad de equipos, la proporción de pruebas en subgrupos poblacionales o el acceso al diagnóstico, sin denominarlos como medidas de cobertura.

La diversidad observada en los estudios refleja la amplitud del propio concepto de cobertura de los servicios de salud que, en un sentido amplio, abarca desde la disponibilidad de recursos hasta la obtención del resultado deseado^(9)^, acercándose en algunos estudios a conceptos como el acceso y la utilización.

Esta imprecisión conceptual, aunque no afecta a las operacionalizaciones legítimas, tiene implicaciones directas para la comparabilidad de los datos: cuando algunos estudios miden la cobertura como la proporción de personas sometidas a pruebas entre quienes presentan síntomas respiratorios, otros como la disponibilidad de equipos y otros como la accesibilidad geográfica, los resultados se vuelven incomparables entre sí. Los diferentes resultados reflejan dimensiones distintas del mismo fenómeno, y equipararlos dificulta tanto el diagnóstico de la situación como la evaluación del impacto de las intervenciones. La ausencia de un marco conceptual común para operacionalizar la cobertura del TRM-TB representa, por lo tanto, una limitación estructural del campo que esta revisión pone de manifiesto.

Esta superposición se reconoce en la literatura sobre sistemas de salud. Penchansky y Thomas definen el acceso como un concepto multidimensional que abarca la disponibilidad, la accesibilidad, la capacidad financiera, la adaptación y la aceptabilidad^(33)^, dimensiones que aparecen de manera fragmentada en las diferentes operacionalizaciones de la cobertura identificadas en esta revisión, lo que refuerza la necesidad de una mayor precisión conceptual en el campo.

Desde diciembre de 2010, la OMS recomienda el uso de la prueba TRM-TB como método diagnóstico primario de la tuberculosis^(2,31)^; sin embargo, se observó que, en dos estudios, el uso de la prueba se vinculó a un resultado negativo en el examen de baciloscopia. Esto pudo haber afectado el resultado de la cobertura en esos lugares, ya que en Guatemala, donde se utilizó este criterio, el TRM-TB por sí solo dio lugar a un aumento del 12,3% en las notificaciones de tuberculosis pulmonar^(21)^. A pesar de su amplia adopción para el diagnóstico de la TB, la baciloscopia tiene una sensibilidad relativamente baja en comparación con el TRM-TB, el cual es capaz de proporcionar resultados mucho más rápidamente y, en consecuencia, permitir el inicio temprano del tratamiento^(3,4,14,32)^.

Un estudio realizado en el estado de São Paulo, Brasil, también señaló que la cobertura de la baciloscopia superó a la del TRM-TB entre las personas que viven con VIH, alcanzando una cobertura del 70%^(28)^, es decir, incluso con la alta cobertura de la TRM-TB, la baciloscopia se ha utilizado de manera complementaria a la prueba, lo que aumenta el número de casos no reportados, sobre todo entre los casos con baja carga bacteriana, como las personas que viven con VIH^(5)^, y también puede dar lugar a la administración de tratamientos sin confirmación bacteriológica.

Se sabe que el riesgo de contraer tuberculosis es 23 veces mayor en las personas que viven con VIH^(34)^; por lo tanto, se refuerza la necesidad de continuar utilizando la prueba TRM-TB en consonancia con estrategias sólidas que fortalezcan la búsqueda activa y el seguimiento de estas personas.

Con base en estos hallazgos, los desafíos mencionados anteriormente en el acceso al diagnóstico mediante el TRM-TB reflejan barreras estructurales y operativas en los servicios de atención, sobre todo para los usuarios coinfectados con tuberculosis y VIH, lo que refuerza la necesidad de estrategias contextualizadas e intervenciones de mejora de la calidad para optimizar la implementación del TRM-TB en esta población específica.

El TRM-TB también tiene la capacidad de detectar bacilos muertos y/o inactivos, por lo que está contraindicado para personas que se encuentran en retratamiento de la tuberculosis^(2)^; sin embargo, en el estudio realizado en Belgorod, Rusia, además del diagnóstico de nuevos casos, el TRM-TB también se utilizó para detectar recidivas de la enfermedad^(14)^, lo que indica un posible uso inadecuado de los recursos, con posibles repercusiones en la gestión de los servicios, los programas y las políticas públicas. Se observaron situaciones similares en el estudio realizado en Corea del Sur, donde se señaló el uso de la prueba en pacientes previamente tratados^(31)^.

Independientemente del criterio adoptado, la cobertura del TRM-TB varió en los diferentes lugares de estudio, y en algunos de ellos se observaron cambios positivos a lo largo de los años. Los resultados corroboran lo que ha venido difundiendo la OMS, según lo cual, en 2024, hubo una variación sustancial en la cobertura de las pruebas rápidas entre regiones y países. Entre las regiones, los mejores niveles de cobertura se alcanzaron en la Región Europea (77%) y en la Región del Pacífico Occidental (70%), mientras que la cobertura más baja se identificó en la Región del Sudeste Asiático (41%). En cuanto a los países, 69 alcanzaron niveles de cobertura de al menos el 80% en 2024, pero en 21 países la cobertura fue inferior al 20%^(2)^. Cabe destacar que la baja cobertura puede ser un indicador de tuberculosis subdiagnosticada, sobre todo la tuberculosis resistente a los fármacos (TB-DR)^(16,22)^.

La Estrategia «End TB» de la OMS establece como meta la eliminación de la tuberculosis como problema de salud pública para 2035, con una reducción del 95% de las muertes y del 90% de la incidencia en comparación con 2015^(35)^. Los datos evidencian que el logro de estos objetivos depende no solo de la disponibilidad del TRM-TB, sino también de la superación de las barreras estructurales, organizativas y conceptuales identificadas en esta revisión.

La baja cobertura del TRM-TB se asoció principalmente a dificultades socioeconómicas y estructurales de los diferentes sistemas de salud; además, se observa la falta de directrices nacionales o regionales para la implementación de pruebas moleculares en los programas de tuberculosis en todo el mundo^(20)^. En 2015, un estudio que hizo un seguimiento de la implementación del TRM-TB en 22 países señaló que, en ese momento, solo Brasil y Rusia financiaban todas las pruebas con recursos nacionales, mientras que la mayoría de los demás países dependían en cierta medida de algunos de los 16 grupos de donantes internacionales identificados^(36)^.

Si bien se debe fomentar el apoyo financiero y de recursos materiales proveniente de terceros países para mejorar el diagnóstico de la tuberculosis, cabe destacar que maximizar los efectos y la sostenibilidad a largo plazo del TRM-TB también depende del liderazgo de las autoridades sanitarias locales, la planificación estratégica, la coordinación con socios técnicos y donantes, así como el monitoreo y la evaluación continuos. Para la implementación a gran escala, es necesario revisar y adoptar algoritmos, políticas, registros, formularios de solicitud y métodos nacionales de monitoreo y evaluación^(30)^.

Se observaron percepciones similares con respecto al acceso a los servicios de diagnóstico de la tuberculosis, incluido el TRM-TB, además de disparidades importantes entre regiones de un mismo país. Un estudio realizado en Nepal sobre las barreras de acceso identificó que las principales dificultades que enfrenta la población están relacionadas con barreras geográficas, como la gran distancia a los servicios, las malas condiciones de las carreteras, la disponibilidad de medios de transporte y los costos asociados al viaje; además, con frecuencia se reporta la falta de personal de salud capacitado en los centros de salud ubicados en áreas periféricas^(37)^.

Los problemas mencionados indican que, además de ser geográficamente inaccesibles, los centros de diagnóstico están constantemente sobrecargados^(25)^. Por lo tanto, las barreras geográficas provocan un efecto en cadena en los indicadores de salud relacionados con la tuberculosis; por ejemplo, las largas distancias hasta los centros dificultan el diagnóstico, la tasa de notificación, el inicio del tratamiento y, en consecuencia, su continuidad^(38)^.

Una revisión sistemática señaló que el escaso conocimiento sobre la tuberculosis entre la población es el principal factor que interfiere en el diagnóstico y el tratamiento, ya que la mayoría de las personas no cuenta con el conocimiento suficiente sobre qué hacer cuando presentan algunos síntomas y cómo obtener la atención y el tratamiento adecuados, especialmente en las zonas rurales^(39)^. Desde la perspectiva de los autores, cabe destacar el factor socioeconómico como una característica importante de la disparidad entre las poblaciones; esto se refleja en el hecho de que la tuberculosis sigue siendo una enfermedad con un elevado estigma social.

También se encontraron variaciones regionales en un estudio realizado en Ghana, donde se observó una disponibilidad inadecuada de equipos y suministros de diagnóstico en el país, con proporciones de centros de diagnóstico significativamente diferentes, con valores que oscilan entre 32 531 personas por unidad de salud en una región y 163 317 por unidad en otras^(38)^.

Es necesaria la presencia de servicios de diagnóstico descentralizados, ya que su ausencia repercute directamente en la acumulación de personas afectadas por la tuberculosis en los principales centros, lo que impide, por lo tanto, el acceso a resultados rápidos de diagnóstico^(39)^. Por lo tanto, es necesario que los recursos procedentes de los programas nacionales contra la tuberculosis no se concentren únicamente en la adquisición de equipos de TRM-TB, sino que, además, se requiera una planificación de gestión para que estas herramientas estén disponibles para la población de manera accesible, con el fin de lograr la equidad en la oferta de servicios de salud.

Además, es importante destacar que la incorporación del TRM-TB puede verse limitada por factores organizacionales y por el mantenimiento de prácticas de diagnóstico previamente establecidas, incluso en contextos con alta capacidad tecnológica^(31)^. Este resultado sugiere que la cobertura de la TRM-TB no depende únicamente de la disponibilidad de la prueba, sino también de la estructura de los servicios, los flujos de atención y la capacitación de los equipos. Por lo tanto, persisten los desafíos para ampliar el uso de la TRM, especialmente en escenarios con alta demanda y heterogeneidad regional.

El presente estudio presenta limitaciones relacionadas con la metodología de la revisión de alcance, como la falta de una evaluación crítica de la evidencia reportada en los estudios incluidos, lo que puede limitar la interpretación de los resultados, además de la heterogeneidad de las diferentes metodologías empleadas. Además, los resultados deben generalizarse con cautela, ya que abordan contextos específicos que presentan realidades y sistemas de salud distintos, así como diferentes incidencias de tuberculosis.

La variación en los criterios de cobertura entre los estudios indica una baja estandarización conceptual en el campo y pone de manifiesto la necesidad de una mayor claridad metodológica y terminológica, especialmente en aquellos que miden aspectos del acceso y la cobertura del TRM-TB, aunque sea de manera secundaria, a fin de evitar la subidentificación de evidencia relevante.

Los resultados de esta revisión, por lo tanto, muestran cómo se ha medido y reportado la cobertura en diferentes estudios y contextos, y qué dimensiones relacionadas con el uso de la prueba se han priorizado en estos escenarios. Aunque estas diferencias metodológicas limitan la comparabilidad directa entre países, ofrecen una perspectiva importante sobre las estrategias locales de implementación de la prueba de detección de la tuberculosis (TRM-TB) y los posibles puntos críticos en las redes de atención a la tuberculosis.

Por lo tanto, esta revisión ofrece una visión general de la cobertura de la TRM-TB e identifica las barreras geográficas, estructurales y socioeconómicas que afectan el diagnóstico; pone de manifiesto la necesidad de una estandarización conceptual de la cobertura y refuerza la importancia de la TRM-TB para el diagnóstico precoz, especialmente en poblaciones en situaciones de vulnerabilidad. Los hallazgos respaldan la implementación de estrategias contextualizadas, la capacitación de equipos, la integración con prácticas tradicionales y la promoción de la equidad, apoyando políticas basadas en la evidencia, la mejora de la atención y la reducción de la subnotificación de la tuberculosis.

## CONCLUSIÓN

Los hallazgos de esta revisión revelaron que, según la literatura analizada, la cobertura del TRM-TB se ha implementado y descrito de maneras distintas en diferentes países y contextos, abarcando desde el porcentaje de personas con tuberculosis evaluadas inicialmente con métodos moleculares hasta indicadores de uso en subgrupos específicos, disponibilidad de equipos y accesibilidad geográfica.

Los estudios indican avances importantes en la incorporación del TRM-TB como método de diagnóstico, especialmente en países con alta carga de tuberculosis, aunque en muchos contextos su uso sigue sin alcanzar las metas internacionales establecidas por la OMS. Los resultados sugieren que la baja cobertura está fuertemente asociada a barreras estructurales y socioeconómicas, a la dependencia de financiamiento externo, a la ausencia de directrices nacionales claras para la implementación de pruebas moleculares y a una descentralización insuficiente de los servicios de diagnóstico.

Para que el TRM-TB alcance su pleno potencial diagnóstico y tenga un impacto positivo en el control de la tuberculosis, se requiere un compromiso renovado de los gobiernos nacionales con la descentralización de los servicios, la disponibilidad y capacitación de los profesionales, así como el desarrollo de políticas locales y regionales. El refuerzo de la financiación, la eliminación de las barreras de acceso y un enfoque más ambicioso para la ampliación sostenida de la cobertura son pasos esenciales para reducir las desigualdades y avanzar en el diagnóstico efectivo de la tuberculosis. Se debe prestar especial atención a las poblaciones en situación de vulnerabilidad, priorizando estrategias que reduzcan el estigma, amplíen el diagnóstico precoz y disminuyan la subnotificación. En el ámbito de la investigación, se recomienda la adopción de criterios estandarizados y alineados con las directrices de la OMS para medir la cobertura del TRM-TB, lo que permite realizar comparaciones entre países y el seguimiento continuo de las metas establecidas.

## Data Availability

Todo el conjunto de datos que respalda los resultados de este estudio se publicó en el propio artículo.
